# Crystalline polymer nanofibers with ultra-high strength and thermal conductivity

**DOI:** 10.1038/s41467-018-03978-3

**Published:** 2018-04-25

**Authors:** Ramesh Shrestha, Pengfei Li, Bikramjit Chatterjee, Teng Zheng, Xufei Wu, Zeyu Liu, Tengfei Luo, Sukwon Choi, Kedar Hippalgaonkar, Maarten P. de Boer, Sheng Shen

**Affiliations:** 1 Department of Mechanical Engineering, Carnegie Mellon University (CMU), Pittsburgh, PA 15213 USA; 2 Department of Mechanical and Nuclear Engineering, Pennsylvania State University, State College, PA 16802 USA; 3 Department of Aerospace and Mechanical Engineering, University of Notre Dame, Notre Dame, IN 46556 USA; 4 Institute of Materials Research and Engineering, Agency for Science Technology and Research, #08-03, 2 Fusionopolis Way, Innovis, 138634 Singapore

## Abstract

Polymers are widely used in daily life, but exhibit low strength and low thermal conductivity as compared to most structural materials. In this work, we develop crystalline polymer nanofibers that exhibit a superb combination of ultra-high strength (11 GPa) and thermal conductivity, exceeding any existing soft materials. Specifically, we demonstrate unique low-dimensionality phonon physics for thermal transport in the nanofibers by measuring their thermal conductivity in a broad temperature range from 20 to 320 K, where the thermal conductivity increases with increasing temperature following an unusual ~*T*^1^ trend below 100 K and eventually peaks around 130–150 K reaching a metal-like value of 90 W m^−1^ K^−1^, and then decays as 1/*T*. The polymer nanofibers are purely electrically insulating and bio-compatible. Combined with their remarkable lightweight-thermal-mechanical concurrent functionality, unique applications in electronics and biology emerge.

## Abstract (web summary)

Polymers compared to structural materials usually have low strength and thermal conductivity. Here the authors show a fabrication method to form bio-compatible crystalline polyethylene nanofibers that exhibit ultra-high strength, thermal conductivity and electrical insulation.

## Introduction

In pursuit of lightweight multi-functional materials for aerospace, automotive, and bio-medical applications, polymers have emerged as a promising platform because they are low-density, easily processable, cheap, and electrically insulating. Yet, bulk polymers are partially amorphous with randomly coiled molecular chains and have defects such as voids, chain ends and entanglements. These defects act as stress concentrators and phonon scattering sites, leading to their low tensile strength (σ_ts_, 20–100 MPa), low stiffness (*E*, 1–5 GPa) and low thermal conductivity (*k*, 0.1–0.4 W m^−1^ K^−1^)^1^. On the other hand, just as a spider synthesizes silk from protein polymer to form a fiber with σ_ts_ similar to high tensile strength steel^2^, polymers can be spun and drawn to form stiff high-strength materials with exceptionally high *k*^3–5^. In particular, at the nanoscale, polymer chains can become highly oriented, and the defects that lower σ_ts_ and *k* can be significantly reduced^6–8^. This leads to simultaneous enhancement in σ_ts_ and *k*, which is different from turbostratic carbon fibers where randomly folded and interlinked graphitic domains enhance σ_ts_ but suppress *k*^9–11^.

Fiber processing techniques such as gel spinning^3–5^, tip drawing^6,7^ and electrospinning^12^ have been developed to enhance crystallinity and chain alignment and thus engineer the thermal and mechanical properties of polymers. Commercial oriented PE gel spun microfibers, with diameters ranging from 10–20 µm, demonstrated enhanced *k* up to 20 W m^−1^ K^−1^ at room temperature (RT)^4^, as well as a σ_ts_ of 3.9 GPa (Dyneema®/ Royal DSM). Electrospinning can produce nanoscale PE fibers with diameters as small as 50 nm, but only moderate crystallinity and alignment were achieved with *k* up to 9 W m^−1^ K^−1^ at 300 K^12^. By optimizing the gel electrospinning process at an elevated temperature, σ_ts_ up to 6.3 GPa on a fiber of 490 nm diameter was achieved^13^. Although tip-drawn PE nanofibers have been reported to have a RT *k* as high as 104 W m^−1^ K^−16^, temperature-dependent thermal transport in such ultra-drawn nanofibers, which is critical for not only elucidating the phonon physics in the nanofibers but also developing their applications in a wide temperature range, remains unknown. Recently, a near theoretical *E* of 312 GPa has been reported in tip-drawn PE nanofibers^7^. However, σ_ts_ of such polymers, which is the limiting factor in many applications, needs to be explored.

Here, we report a fabrication method that can consistently produce polyethylene (PE) nanofibers with diameters ranging from 10 to 100 nm. We measure *k* over a broad temperature range from 20 to 320 K. We find that *k* increases with temperature, following an unusual *T*^1^ trend below 100 K eventually peaking around 130–150 K, and reaching a value as high as 90 W m^−1^ K^−1^. Thereafter, *k* decays as 1/*T*. Measurements show ultra-high σ_ts_, 11.4 ± 1.1 GPa, for our crystalline PE nanofibers. To the best of our knowledge, this is the highest σ_ts_ measured for any polymer based fibers, including PE fibers (Dyneema®/ Royal DSM and Spectra®/ Honeywell), aramid fibers (Kevlar®/ DuPont), carbon fibers and composite fibers^13–16^.

## Results

### Two-step fabrication of crystalline PE nanofibers by local heating

**Fig. 1:**
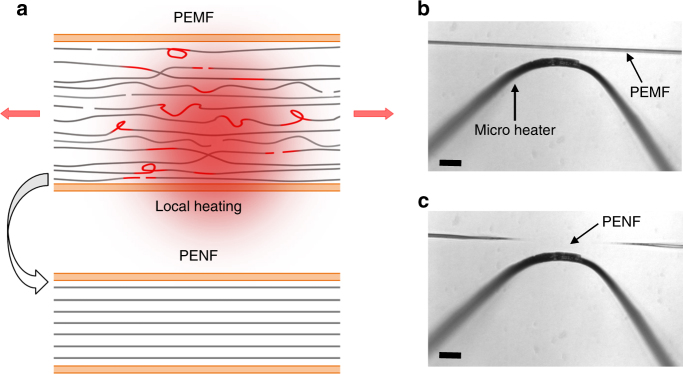
Fabrication of a PE nanofiber (PENF). **a** Idealized schematics of localized drawing with a microheater to fabricate PENF from a PE microfiber (PEMF). Arrows indicate drawing directions whereas red lines highlight the defects in PEMF. **b-c** Optical micrograph before and after localized drawing (in **c**, nanofiber not resolved optically). Scale bars, 20 µm

The fabrication of PE nanofibers, PENFs, was conducted in two steps where a microfiber, PEMF, was tip drawn from a PE/decalin gel and then further stretched to the nanometer scale by localized heating in air (see Methods and Supplementary Note 2). As shown in Fig. 1, a small segment of a pre-stressed PEMF was placed near a simple micro heater, and locally heated near the melting point. The pre-existing tensile stress quickly stretched the fiber (see Supplementary Movie 1) to a diameter in the range of 10–100 nm, which could be controlled by tuning the pre-existing stress or the heater temperature. Per the schematic in Fig. 1a, amorphous regions are stretched and recrystallized, leaving a nearly defect-free aligned crystalline PENF.

The local heating method developed in this work has a number of advantages over the regular heating method, where the whole fiber is heated and stretched over an oven or hot plate until reaching a nanoscale diameter^6,7^. The *k* of PE is directly correlated with the crystallinity and molecular alignment along the fiber axis of the sample. The average extension of molecules increases with the strain rate^17^. With the drawing by local heating, a strain rate up to 1400 s^−1^ can be achieved (see Supplementary Note 2) compared to ~1000 s^−1^ in gel electrospinning^13^ and ~1 s^−1^ in gel spinning^5^. Another important aspect is the relaxation of molecules, which competes with stretching. In local heating, the fiber is rapidly quenched to minimize the relaxation. The resulting high degree of alignment, coupled with high crystallinity, leads to the improved σ_ts_ and *k*. The fiber drawing by local heating yields a segment of nanofiber (~100 µm long) that spans between microfibers. Using the microfiber segments for tracking, the nanofiber can be precisely manipulated onto test platforms. In addition, the fabrication yield is much higher with local drawing. In regular drawing, hot air flow rising from the hot plate/oven makes the fiber stretching susceptible to local hot spots. A fiber with various hot spots is more likely to have different local stress and strain, increasing the likelihood of premature breakage.

### Crystal structure of PE nanofibers

**Fig. 2:**
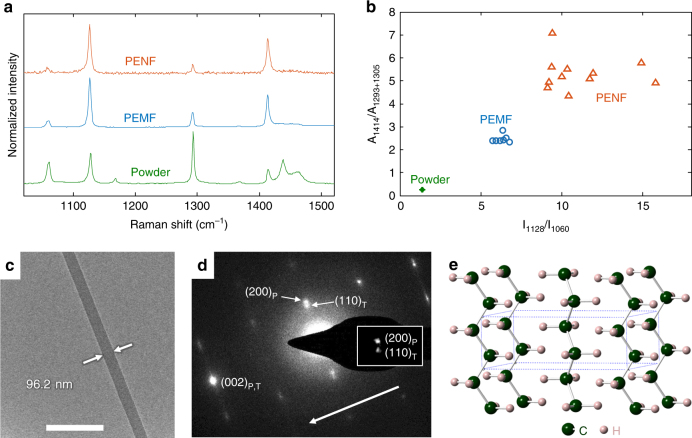
Morphological and structural characterizations. **a** Micro-Raman spectra of PE powder, PEMF and PENF where the data is normalized to the highest peak of each spectrum. **b** Ratio of integral areas of Raman band 1414 cm^−1^ to Raman bands 1293 cm^−1^ and 1305 cm^−1^ vs. intensity ratio of Raman bands 1128 cm^−1^ to 1060 cm^−1^. **c** TEM micrograph of a PENF. **d** SAED pattern of the PENF in **c** Inset shows the distinct diffraction spots of (200) plane of parent crystal (subscript P) and (110) plane of twinned crystal (subscript T). Arrow indicates the drawing direction. **e** Orthorhombic crystal structure of PE. Scale bar, 500 nm

To characterize the PENF crystal structure, we employed micro-Raman and low-dose transmission electron microscopy at cryogenic temperatures (cryo-TEM). The micro-Raman was used to understand the orientation and crystallinity of PE during different stages of drawing. A qualitative measure of molecular orientation can be obtained from the peak intensity ratio of 1128 cm^−1^ and 1060 cm^−1^ Raman bands^18^. Similarly, the crystallinity can be measured by the ratio of the integral areas under Raman bands of 1414 cm^−1^ to 1293 cm^−1^ and 1305 cm^−1^, where Raman band of 1414 cm^−1^ and Raman bands of 1293 cm^−1^ and 1305 cm^−1^ correspond to the orthorhombic crystal and an internal standard independent of chain conformation, respectively^18^. Figure 2a shows typical Raman spectra of PE powder, PEMF and PENF. The spectra show that the area under the 1414 cm^−1^ band increases from powder to PEMF and then PENF indicating that the amorphous phase is significantly reduced and crystallization is promoted (see Supplementary Note 2 for crystallinity of PE powder). In addition to crystallinity, molecular orientation also increases from powder to PEMF and then PENF (Fig. 2b).

The structural and morphological properties of PENF were further characterized using low-dose cryo-TEM. Figure 2c shows a TEM micrograph of a typical PENF sample. Selected area electron diffraction (SAED) patterns (Fig. 2d) with an orthorhombic crystal structure (Fig. 2e) of crystalline PE with lattice constants *a* = 7.301 Å, *b* = 4.893 Å and *c* = 2.550 Å were indexed. These constants agree well with literature values from experiments and molecular simulations^19–21^. The arrow indicates the drawing direction, which is along the *c*-axis, and shows that (001) remains along the fiber axis. During the highly localized heating and drawing, the PEMF is stretched at a high strain rate while undergoing plastic deformation, which can occur via slip, twinning or martensitic phase transformation^22,23^. Among the principal {110} and {310} twin planes that can be activated in PE, {310} twinning may occur at high strain under which the morphology changes from lamellar to fibrillar^24^. The SAED pattern (Fig. 2d) indicates that in oriented PENF {310} twinning occurs. Because the twinning involves rotations about the *c*-axis (54°) and the properties in this work are measured along that axis, the effects are expected to be negligible. The presence of a monoclinic phase was also observed in SAED pattern but its volume fraction is small (see Supplementary Fig. 8).

### Thermal transport in crystalline PENF

**Fig. 3:**
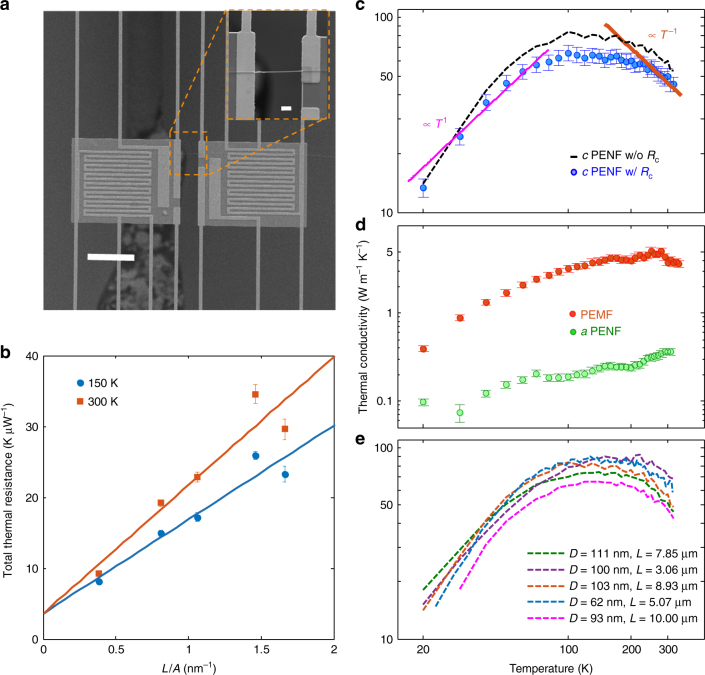
Thermal characterization of PENF. **a** A PRT based micro-device with the heating and the sensing islands. Inset shows a PENF bridging the islands. **b** Total measured thermal resistance vs. *L*/*A* of crystalline (*c*) PENFs at 150 K and 300 K where the *y*-intercept represents 2*R*_c_. The error bars show the mean and standard deviation values of 250 measurements. **c** *k*(*T*) of a PENF. Blue circles are measured *k* of a *c*-PENF, black dotted lines are after adjusting for 2*R*_c_. Orange line is 1/*T* trend of *c*-PENF at high temperatures and pink line is *T*^1^ trend at low temperatures. The error bars are obtained using error propagation in 2*R*_c_ and measured diameter (see Supplementary Note 4 for detailed uncertainty analysis). **d** *k*(*T*) of PEMF and amorphous (*a*) PENF. Red circles are PEMF before local drawing, and green circles are *c*-PENF amorphized due to exposure to FIB. **e** *k*(*T*) of all reported samples after adjusted for 2*R*_c_. Scale bars, 20 µm and 2 µm (inset)

We measured the temperature-dependent *k*(*T*) of PENF in high vacuum (2 × 10^−7^ Torr) using a suspended platinum resistance thermometer (PRT) microdevice (see Fig. 3a), which consists of heating and sensing measurement islands^25–27^. To achieve a low thermal contact resistance, a platinum or graphite coating deposited by electron or focused ion beam (FIB) has been extensively used in literature^28,29^. However, these methods cannot be applied in our case due to the sensitivity of PENFs to high energy electron/ion beams, both of which severely damage the crystallinity and molecular orientation of the samples^30^ (see Fig. 3d and Supplementary Fig. 13). To enhance the thermal contact between PENF and the device, we exploited a technique we call capillary-assisted adhesion of PENF by first placing an isopropanol (IPA) drop on top of the thermal device. Surface tension from the evaporating liquid then pulls the PENF into intimate contact with the pads.

The equivalent PENF thermal circuit can be expressed as a series of thermal resistances, namely intrinsic thermal resistance of the nanofiber *R*_s_, thermal contact resistance between the nanofiber and the heating island $R_{{\mathrm{c}},{\mathrm{hi}}}$ and the sensing island $R_{{\mathrm{c}},{\mathrm{si}}}$. The heating and sensing islands have the same geometrical and material properties and undergo the same contact mechanism, so we can assume that $R_{{\mathrm{c}},{\mathrm{hi}}} \approx$ $R_{{\mathrm{c}},{\mathrm{si}}} \approx R_{\mathrm{c}}$. The measured *R*_tot_ is the total thermal resistance of this circuit. Therefore,

1 $$R_{{\mathrm{tot}}} = R_{\mathrm{s}} + 2R_{\mathrm{c}} = \frac{L}{{kA}} + 2R_{\mathrm{c}}$$

where *L* is the gap length between the heating and the sensing islands, *A* is the cross-sectional area of the fiber and 2*R*_c_ is the total thermal contact resistance. Assuming similar *k* for the samples with similar diameter, we estimate 2*R*_c_ by extrapolating the linear fit between *R*_tot_ and *L*/*A* to y-intercept. The linear fits (Fig. 3b) at 150 K and 300 K show that 2*R*_c_ is 3.6 × 10^6^ K W^−1^ and is approximately temperature-independent above 100 K^31^. This value is consistent with (2*R*_c_ ≈ 4 × 10^6^ K W^−1^) predicted using a line-contact model (see Supplementary Fig. 14). Figure 3c shows the *k*(*T*) of a typical nanofiber, which increases as ~*T*^1^ and reaches a maximum of ~90 W m^−1^ K^−1^ at 130 K–160 K. It then decreases as 1/*T* on further increasing the temperature. RT values were 50–70 W m^−1^ K^−1^ (see Fig. 3e), which is on the same order as those of tip drawn nanofibers^6^. This is a significant enhancement (10–14×) using local drawing as the PEMF RT *k* is 5 W m^−1^ K^−1^ (see Fig. 3d). However, PENFs with similar diameter show scatter in RT *k* values which may be because of the variation in crystallinity and orientation among the nanofibers as shown in Fig. 2b. Recent studies on ultra-drawn PEMF and films have also obtained *k* around 50–60 W m^−1^ K^−1^ at RT^32–34^. After careful characterization on the cross section of the PENFs, we observed that samples with diameters below 150 nm generally have a non-circular cross-section (See Supplementary Fig. 21, where we provide extensive documentation detailing how this was characterized), in which the measured height is about 1.5 times smaller than the measured width. We assumed the fibers to have a cylindrical shape and used the measured width for calculating *k* of PENFs from the measured thermal conductance. Hence, the *k* reported in Fig. 3c and e should be taken as a lower bound.

**Fig. 4:**
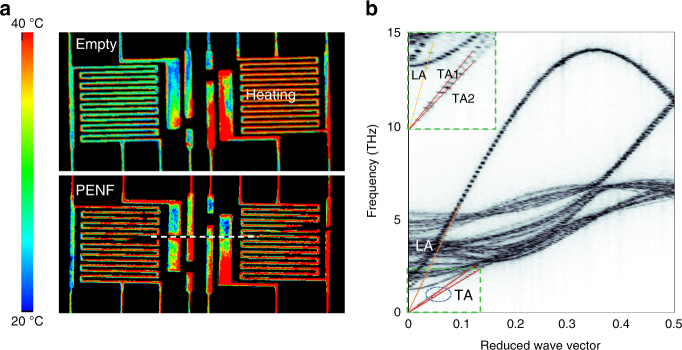
Thermal transport in PENF. **a** Thermoreflectance imaging of heating island (right) and sensing island (left) without a nanofiber and a PENF respectively, in laboratory air. Dotted white line is the PENF position. **b** Phonon dispersion relation of the acoustic branches along the chain backbone calculated from the trajectory of MD simulations using two-dimensional Fourier transform. The contour is the raw data from calculation and colored lines are linear fits. The inset shows the acoustic branches close to the Brillouin zone center

To directly demonstrate the heat transfer along the PENF we used high-resolution thermoreflectance imaging (Microsanj NT-210B) to precisely map the temperature distribution of the micro thermal device in Fig. 4a, in which the island on the right-hand side was electrically heated. We compared the following two cases for the same batch of devices at the same heating power in laboratory air. In the absence of a nanofiber bridging the two islands, the left island was heated negligibly due to the low thermal conductivity of the air gap (Fig. 4a). In the case of our fabricated PENF with a diameter of ~500 nm (Fig. 4a), the left island of the micro device was dramatically thermalized because of the extremely high thermal conductivity (~70 W m^−1^ K^−1^). This experiment demonstrated that the PENF can potentially be used as efficient heat spreaders for microelectronics. Importantly, compared with existing nanoscale heat spreaders like carbon nanotubes and graphene, the PENF have a practical advantage because they are purely electrically insulating.

While it is well understood that the 1/*T* relation of *k* at high temperatures is due to anharmonic phonon scattering in crystals, the *T*^1^ relation at low temperatures is uncommon in crystalline materials^28,35^. To understand this unusual *k*(*T*) trend of PENFs, we performed atomistic simulation to analyze the phonon properties in a perfect PE crystal with extended chains (see Methods). We first calculated the phonon dispersion relation along the chain backbone from molecular dynamics simulations (Fig. 4b). Similar to common 3D crystals, there are three acoustic phonon branches, with each being linear close to the Brillouin zone center. If we consider the relaxation time approximation for the phonon Boltzmann Transport Equation, we can use the following equation to express the thermal conductivity:

2 $$k = \mathop {\sum }\limits_{{\mathbf{q}},\alpha } c_{{\mathbf{q}},\alpha }v_{{\mathbf{q}},\alpha }l_{{\mathbf{q}},\alpha }$$

where *c*_**q***,α*_, *v*_**q***,α*_, and *l*_**q***,α*_ are respectively specific heat, group velocity and mean free path of phonon with wavevector **q** and polarization *α* along the chain backbone direction. This is a scalar *k* along the chain backbone direction which can also be deduced from the more general tensor form^36^. At low temperatures, where the anharmonic phonon scattering is weak, the mean free path is usually limited by extrinsic scattering mechanisms, such as defect and boundary scattering. In our case, these can be from polymer chain ends or other defects along the chain backbone, such as segment rotation, which are much stronger scattering centers than point defects^21,37^. Under such a condition, we assume that the mean free path is the same for all phonons, which is also a common assumption made for 3D crystals at low temperatures^38^. As a result, *k*(*T*) is purely from the heat capacity. For the linear phonon branches in a 1D system, *k* should scale as *T*^1^^38^. Thermal transport in aligned PE chain bundles is highly anisotropic and is dominated by the chain backbone since the inter-chain vdW interactions are much weaker than the covalent bonding along the backbone. However, phonons with wavevectors not strictly aligned along the chain backbone direction can still contribute to *k* though in a much smaller proportion. This 3D effect of phonon dispersion can result in *k* scaling slightly greater than *T*^1^ at the low temperature limit, which can be seen in Fig. 3c. At the high temperature end, the experimental data also matches well with results from early MD simulations^37^ where non-equilibrium MD was used to calculate the *k* of crystalline PE. The high temperature data can be reasonably fit by a 1/*T* relation.

### Ultra-high σ_ts_ of crystalline PENF

**Fig. 5:**
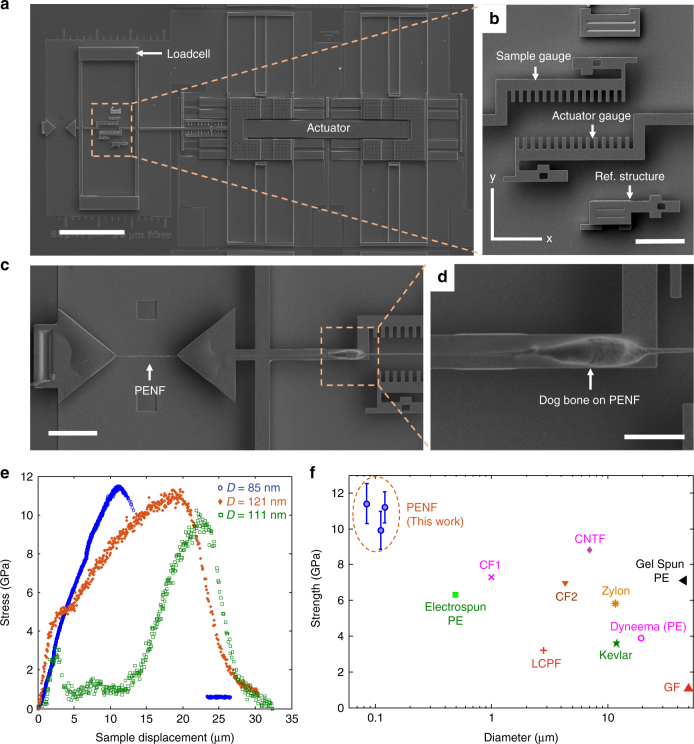
Mechanical characterization of PENF. **a-d** SEM images of MEMS device used for tensile test of PENF. **b** Gauges for pattern matching to obtain sample displacement and loadcell extension. **c** A PENF gripped between two pads. **d** Dog bone shape on a PENF which provides additional grip due to mechanical locking. High contrast at the edges of dog bone is from charging of cured epoxy. **e** Stress-displacement curve. **f** σ_ts_ of PENF compared to other fibers such as electrospun PE^13^, CF1^14^ (Carbon fiber 1), CF2 (HexTow®/ Hexcel), CNTF^41^ (Carbon nanotube fiber), PE^42^ (Gel-spun PE fiber), Zylon®/ Toyobo, LCPF (Liquid crystal polymer fiber, Vectran^TM^/ Kuraray), Kevlar®/ Du Pont, Dyneema®/ Royal DSM, and GF^16^ (Graphene fiber). The error bars in PENF are calculated using uncertainty analysis (See Supplementary Note 5). Scale bars, 200 µm (**a**), 25 µm (**b, c**), and10 µm (**d**)

The σ_ts_ of individual nanofibers was tested in tension using a microelectromechanical systems (MEMS) based device with an on-chip actuator that is a micro stepper motor with 60 nm step size and large in-plane force (up to 1 mN)^39,40^. The actuator displacement range is >100 µm and can apply large strain on a 30 µm nominal gauge length nanofiber. The metrology based on optical microscopy has a displacement resolution of 4 nm and a force resolution of 25 nN under a ×50 objective (see Methods). The MEMS device (Fig. 5a–d) is fabricated with an on-chip actuator and a loadcell spring in series. The sample is gripped between the loadcell and a pad rigidly connected to the substrate. When an actuator takes a step to the right, it pulls the loadcell and the specimen. Since the loadcell and the specimen are in series, the force on the sample can be obtained from the stiffness and the extension of the loadcell. Similarly, the specimen displacement can be obtained from the sample gauge displacement. After the mechanical measurement, the diameter and the length of the fiber were measured by SEM. A stress-displacement curve was generated, from which the σ_ts_ was obtained.

In the tensile tests, it is challenging to grip the PENFs using adhesive because of the low surface energy of PE^43^ (31 mJ m^−2^), and slip occur before they are tested to failure. We thus designed a dog bone shaped nanofiber (Fig. 5c, d) to provide additional mechanical locking (see Supplementary Note 6). However, even then, some slip still occurs, and thus the *E* cannot be characterized accurately in our method. It should be noted that in Fig. 5e, the sample with *D* = 111 nm slips after the stress exceeds 3 GPa, then becoming taut until the dog bones provide the necessary grip.

The σ_ts_ of 11.4 ± 1.1 GPa was obtained for the nanofiber with diameter 85 nm. To our knowledge, this is the highest measured σ_ts_ for any polymer fiber reported including carbon fiber, Zylon®, Kevlar® and nylon fibers^13–15,44^ (Fig. 5f). Even though the theoretical C–C bond strength (for chain scission) can be as high as 20 GPa, PE strength is limited by slip that initiates at chain ends (6–8 GPa)^45^. The failure mode is a combination of chain slip and chain scission. A significantly minimized chain end density within the test length due to the local heating based fabrication technique and short gage length (30 µm), comparable to the length of the PE molecule (~30–55 µm for ultra-high molecular weight PE), explains the high strength. In addition, the diameter is not uniform over a length of 30 µm (see Supplementary Fig. 22). For mechanically tested samples, the narrow region along the sample used for calculating the σ_ts_ spans less than 1 µm (see Supplementary Fig. 19). As this is much smaller than the length of a PE molecule, the likelihood of chain ends in the region is negligible. Therefore, these regions can sustain substantially larger stress than those limited by chain ends.

Two distinct failure mechanisms were observed when tested with low (5.4 N m^−1^) and high (50.8 N m^−1^) stiffness loadcells. The sample tested with the low stiffness loadcell exhibited ductile failure (Fig. 5e, *D* = 85 nm), whereas that with the high stiffness loadcell experienced extreme necking, where the diameter was reduced to less than 10 nm (see Supplementary Fig. 20). This is because of the elastic strain energy $U = (1/2)k_{\mathrm{L}}\delta _{\mathrm{L}}^2 = (1/2)F^2/k_{\mathrm{L}}$ stored in the loadcells. For a given load *F*, higher elastic strain energy is stored in the lower stiffness loadcell. For small *k*_L_ (5.4 vs. 50.8 N m^−1^), *F* drops slowly during displacements after the σ_ts_ is reached, increasing the likelihood of instability.

## Discussion

In summary, we have demonstrated highly aligned and crystalline PENF with diameters of 10–100 nm fabricated by a localized heating and drawing method. Both SAED and micro Raman analyses clearly verify the high crystallinity and molecular alignment. Values of *k*(*T*) from 20 K to 320 K are reported, where *k* reaches up to 90 W m^−1^ K^−1^ at 130 K. This suggests that PENF can be used as an efficient heat transfer material for cryogenic purposes particularly where an electrically insulating material is concurrently desired^46^. At low temperatures, *k* increases as ~*T*^1^, which stems from the one dimensionality of phonon transport and extrinsic scattering (e.g., defects, boundary). Measurement of σ_ts_ using a MEMS device demonstrates that these nanofibers have ultra-high σ_ts_ up to 11.4 ± 1.1 GPa. This is the highest σ_ts_ reported for any polymer fiber or composite.

The values we obtained for σ_ts_ and *k* are critically dependent on the cross-section area, which is challenging to measure at this scale. As stated above and detailed in the Supplementary Note 7 (Supplementary Fig. 21), the cross-sections are non-circular. However, when calculating σ_ts_ and *k* of PENF from the measured force and thermal conductance we assumed that the fibers have a circular cross-section, and used the larger dimension. Hence, the *k* reported in Fig. 3c and e and σ_ts_ reported in Fig. 5e and f should be taken as a lower bound for PENF.

The polymer nanofibers with lightweight-thermal-mechanical functionality could find general applications in aerospace and automotive systems, where a high strength-to-weight ratio (Supplementary Note 1) is desirable. With the multifunctionality, the polymer nanofibers are purely electrically insulating and bio-compatible, which opens up unique applications in electronics and biology. These nanofibers provide highly effective heat removal in electronic systems, for example, heat sinks for electronic packages. Furthermore, integrating local heating as a secondary process on gel spun microfibers could potentially realize making larger scale PE nanofibers.

## Methods

### Localized heating/drawing to fabricate PENF

In this method, we first produce PE/decalin gel. Then, a sharp glass tip (~10 µm) is used to pull a PEMF at 90  °C. Tip drawing of PEMF from PE/decalin gel at an elevated temperature results in the unfolding of the chain lamellae into micro fibrils that are pulled taut between entanglements^6^. PEMF was then quenched to RT to reduce the relaxation of the aligned molecules. It was kept taut and attached to a sample collector, which is a bulk micromachined rectangular silicon frame with a square hole (see Supplementary Fig. 3). The pre-stressed PEMF on the sample collector was locally heated and stretched to nanoscale diameter using a home built microheater, which is a sharply bent tungsten micro wire with an etched tip. When a small segment of the PEMF was placed in proximity to the tip of the heater and heated close to the melting point, the pre-existing tensile stress immediately stretched the PEMF to a nanoscale diameter (see Supplementary Movie 1). A section of 80–100 µm long nanofiber was usually obtained in a PEMF using this two-step method. The eventual diameter of the nanofiber can be controlled by tuning the pre-existing stress or the temperature of the heater.

### Micro Raman characterization

We used a Reinshaw InVia Raman Microscope with a 532 nm laser. The sample was aligned using a low laser power of 2.5 mW to reduce the irradiation damage prior to characterization. An incident laser with 25 mW power was focused with a ×50 objective onto a PE fiber suspended across a sample collector. A backscattered Raman spectrum was accumulated for 60 s at room temperature. The sample undergoes minimal structural damage during the characterization, which is verified by overlapping three separate Raman spectra collected at the same spot of a PENF sample (see Supplementary Fig. 10). The Raman spectra were collected with a spectral resolution of 0.9 cm^−1^ between 1000 cm^−1^ and 1500 cm^−1^.

### Low dose cryo-TEM

Structural characterization of crystalline polymer is challenging under an electron beam as the radiation damages the sample crystallinity, which leads to fading and evolution of the diffraction spots into a ring pattern. The damage can be observed on a PE crystal at a dose as low as 25 × 10^−4^ C cm^−2^, and complete loss of Bragg spots occurs at a critical dose of 0.01 C cm^−2^ at an accelerating voltage of 100 kV^30^. The critical dose can be increased by reducing the temperature; in PE crystals it can be increased by a factor of 2.5 by cooling the sample from RT down to 100 K^30^. To reduce the electron beam damage and characterize morphological and structural features, we used low dose cryo-TEM. The sample was cooled to 100 K using a liquid nitrogen cooled sample holder. The sample was inspected at a low dose of 8 × 10^−5^ C cm^−2^, whereas selected aperture electron diffraction (SAED) patterns were obtained at a dose close to the critical dose. Bright field images and SAED patterns were obtained at 80 kV.

### Phonon dispersion calculation

To obtain the phonon dispersion of aligned PE chain bundles, we performed MD simulations to generate the trajectory. The condensed-phase optimized molecular potentials for atomistic simulation studies (COMPASS)^47^ all-atom potential is used to model the PE and a timestep of 0.5 fs is employed. In the simulation domain, there are 4 chains in the unit cell and 400 CH_2_ segments in each chain with periodic boundary condition applied in all three spatial directions. After structural minimization, the simulation first runs at 2 K in the NVT (constant volume and temperature) ensemble, and then runs in the NVE (constant volume and energy) ensemble for 50 ps to produce the trajectory. The low temperature is chosen to reduce the noise and increase the contrast of the dispersion relation. During the NVE run, the velocity of every backbone carbon atom is recorded every 5 fs in a two-dimensional matrix. Then, a two-dimensional Fourier transform of the atomic velocity of one carbon atom in the unit cell is performed:

3 $$\Phi (\omega ,{\mathbf{k}}_{\mathrm{v}}) = \sqrt {\mathop {\sum }\limits_\alpha ^3 \left| {\frac{1}{N}\mathop {\sum }\limits_{n = 0}^{N - 1} e^{{\mathrm{i}}\frac{n}{N}{\mathbf{k}}_{\mathrm{v}}}{\int} {v_\alpha (n,t)e^{ - {\mathrm{i}}\omega t}\mathrm{d}t} } \right|^2} ,\alpha = x,\;y,\;z$$

where $v_\alpha (n,t)$ is the atomic velocity, *ω* is the the frequency, **k**_v_ is the wavevector, *n* is the index of repeating unit along chain direction, and *N* is the number of the repeating unit. Next, the phonon dispersion relation is calculated by averaging the two-dimensional Fourier transform results of all the backbone atoms in the unit cell. The obtained dispersion plot, which is essentially a two-dimensional contour, is digitized and fitted with a polynomial (see Fig. 4b). We have also used the same method to calculate the dispersion of a standalone single PE chain, and the obtained dispersion relation agrees well with other calculations in the literature^48^. At the low temperature limit, where anharmonic phonon scattering is weak, we assume that extrinsic scattering will cause the phonon mean free path be the same for all phonon modes.

### Stress and displacement measurements

After the sample was mounted and aligned onto the MEMS device, measurements were carried out on a custom designed probe station equipped with a long working distance microscope on a vibration isolation table at RT. The actuator was actuated at 1 step per second while load cell displacements were optically imaged with a ×50 objective (NA = 0.55) using a charge coupled device (CCD) camera at 1 frame per second and stored for data analysis. Images were analyzed to measure load cell displacements with resolution of 4 nm using sub pixel pattern matching. Load cell displacement measurements were used to calculate force and sample displacement. The force exerted on the sample is $F = k_{\mathrm{L}} \times \left( {x_{{\mathrm{actuator}}\;{\mathrm{gauge}}} - x_{{\mathrm{sample}}\;{\mathrm{gauge}}}} \right)$. After the diameter (*D*) was measured using SEM, stress was calculated as $\sigma = 4F/({\mathrm{\pi }}D^2)$. Sample displacement was obtained as $u = \left( {x_{{\mathrm{sample}}\;{\mathrm{gauge}}} - x_{{\mathrm{ref}}\;{\mathrm{structure}}}} \right)$.

### Sub pixel pattern matching for nanoscale resolution using optical microscopy

Sub pixel pattern matching is used to obtain nanoscale resolution of displacement from optical images^49,50^. Direct measurement is diffraction limited, however, this can be circumvented by comparing two images and obtain displacement resolution smaller than a pixel. In sub-pixel pattern matching, a predetermined 2D pattern of a first image is iteratively searched in an expected window in subsequent images. Pattern positions (*x*, *y*, and *θ*) with the highest match score are recorded. Sub pixel displacement resolution is obtained by interpolating a pixel (bilinear here) on discrete structures such as edges or lines where a sharp contrast is achieved. Each pixel has a greyscale value from 2^*n*^ possible values where *n* is the number of bits per pixel (*n* = 8 in our case). A perfect edge with white (1) on one side and black (0) on other in a pixel could be interpolated with 2^*n*^ intensity values. In an ideal scenario with no noise and perfect contrast, a resolution of pixel/2^*n*^ can be achieved. This would give 0.5 nm displacement resolution for an objective of ×50 (pixel length is 121 nm). Considering noise, we achieved pixel/30 i.e., ~4 nm resolution (1 standard deviation) as aided by a large number of well-defined edges as provided here by the comb structures.

### Data availability

The data that support the findings of this study are available in Supplementary Information and from the corresponding authors upon reasonable request.

## Electronic supplementary material

Supplementary Information

Description of Additional Supplementary Files

Supplementary Movie 1
