# Supplementary material for: Crystalline polymer nanofibers with ultra-high strength and thermal conductivity
Source: Nat Commun. 2018 Apr 25;9:1664. doi: 10.1038/s41467-018-03978-3 (PMC5916895; doi:10.1038/s41467-018-03978-3)
Supplement: Supplementary file 1 — Supplementary Information [file 41467_2018_3978_MOESM1_ESM.pdf]

Supplementary Information

Crystalline Polymer Nanofibers with Ultra-high Strength and Thermal Conductivity

Ramesh Shrestha[1], Pengfei Li[1], Bikramjit Chatterjee[2], Teng Zheng[3], Xufei Wu[3], Zeyu Liu[3], Tengfei Luo[3], Sukwon Choi[2], Kedar Hippalgaonkar[4], Maarten P. de Boer[1, *], Sheng Shen[1, *]

[1]Department of Mechanical Engineering, Carnegie Mellon University (CMU), Pittsburgh, PA 15213 USA

[2]Department of Mechanical and Nuclear Engineering, Pennsylvania State University, PA 16802 USA

[3]Department of Aerospace and Mechanical Engineering, University of Notre Dame, Notre Dame, IN 46556

[4]Institute of Materials Research and Engineering, Singapore 117602, Singapore

[*]Corresponding authors. E-mail: sshen1@cmu.edu; mpdebo@andrew.cmu.edu.

Supplementary Note 1: Ashby map of specific tensile strength and thermal conductivity

Specific strength vs thermal conductivity

Specific tensile strength vs thermal conductivity, k, of polyethylene nanofiber (PENF) is compared with other existing materials available in CES EduPack software (CES Edupack/ Granata Design Ltd.). Supplementary Fig. 1 shows that PENF has higher specific strength than state-of-the-art values of any polymer material[1]. Even though it is lower than carbon nanotube (CNT) and graphene in both specific strength and k, it has a practical advantage where electrically insulating material is needed.

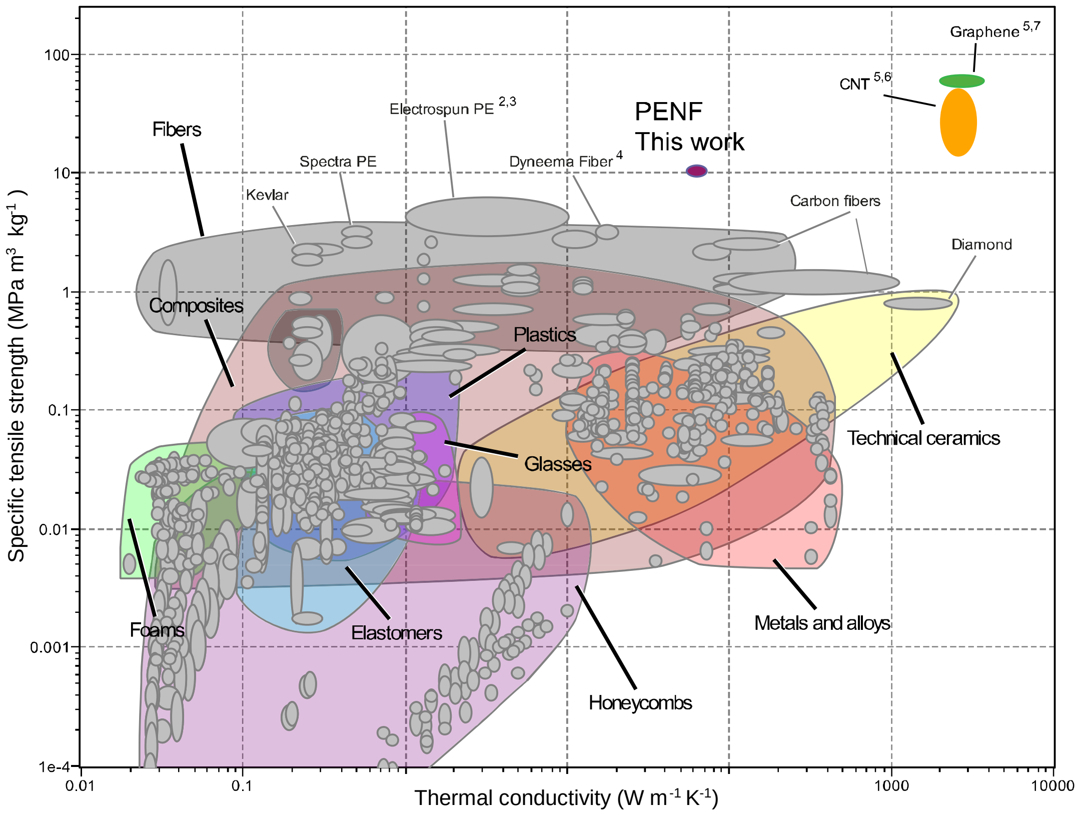

Supplementary Figure 1: Ashby chart of specific tensile strength vs k of materials from CES Edupack (2017) apart from those with reference[2–7].

Supplementary Note 2: Details about the fabrication and positioning of PENF

Fabrication of polyethylene microfiber from the gel

In this fabrication method, we first produce the PE gel. To make the PE gel, we used 0.8 wt % ultra-high molecular weight polyethylene (UHMWPE) powder (average molecular weight 3 – 6 × 10^6 g mol^{-1} purchased from Sigma Aldrich) and mixed it with a decalin solvent. The mixture is heated on a hot plate to 145 ˚C. It becomes transparent and viscous as the PE powder dissolves in the solvent. A glass rod is used to constantly stir the solution. To avoid oxidation and subsequent molecular degradation, this process is carried out inside an argon filled glove box. The solution is then quenched in a room temperature water bath, and the gel forms.

After gel preparation, a two–stage tip drawing method is used to form a microfiber, PEMF. A 5 mm × 5 mm silicon chip with a thin film heater attached on the backside is used to heat the gel to 120 – 130 ℃. A hot plate placed 1 cm below the silicon chip heats the overhead air to 90 ℃ to prepare for hot-stretching. The fabrication setup is shown in Supplementary Fig. 2. The translucent gel turns to a transparent solution as it is heated to 130 ℃. As the solution turns clear, a sharp glass tip (10 µm) is used to draw a short length (several hundreds of microns) of PEMF. The fiber undergoes stress-induced crystallization. During crystallization, the decalin evaporates, aided by the convective current from the hot plate. Decalin syneresis further facilitates crystallization[8]. The PEMF is then further drawn to a length of 1 cm after which it is quenched to room temperature to minimize relaxation of the extended chain.

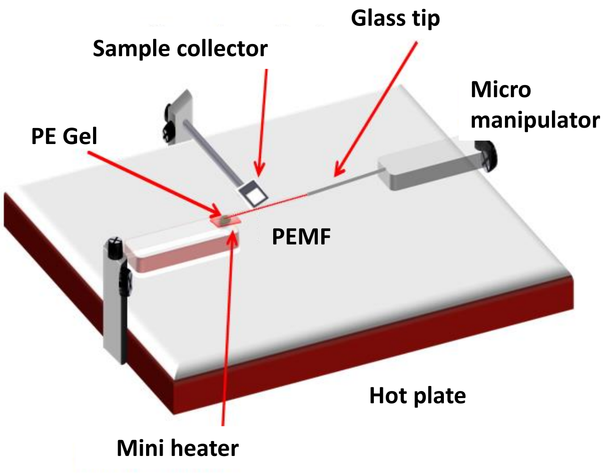

Supplementary Figure 2: Schematic of fabrication setup of tip drawing.

After the drawing is complete, PEMF is placed on a sample collector as shown in Supplementary Fig. 3a, where it remains under tensile stress. The collected PEMF is locally heated using a micro heater to make nanofibers as shown in Supplementary Fig. 3b and described in main text.

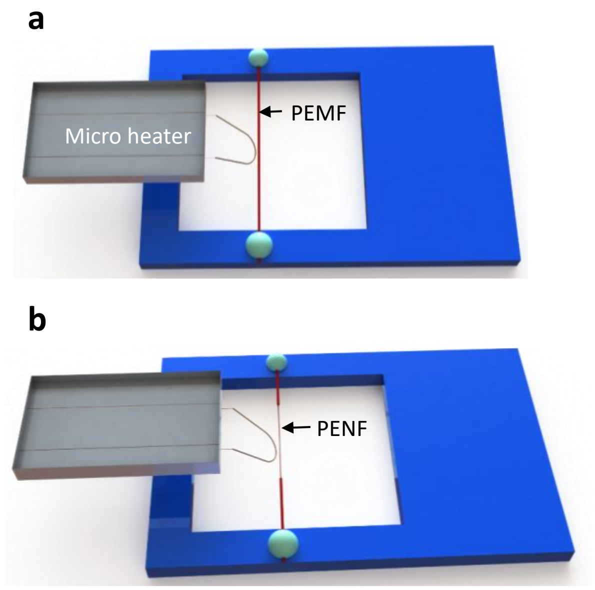

Supplementary Figure 3: Schematic of local drawing of PE microfiber (PEMF) to PE nanofiber (PENF). **a**) Pre-stressed PEMF on sample collector. **b**) PENF formed on the PEMF using local drawing.

Temperature profile of the PEMF during local drawing

Here, we estimate the maximum temperature attained by the fiber during local heating. First, we simulated the temperature profile of the micro heater in ANSYS. Electrothermal modeling with a potential bias 0.7 V was applied to the heater, same as in our experiment. Both convection and radiation heat losses were considered in the simulation. In microsystems, the boundary layer gets smaller, thus, heat transfer coefficient (h) greatly increases. Here, we used h = 1000 W m^{-2} K^{-1}, emissivity = 0.1 in our simulation[9]. Supplementary Fig. 4 shows the simulated temperature profile of the heater.

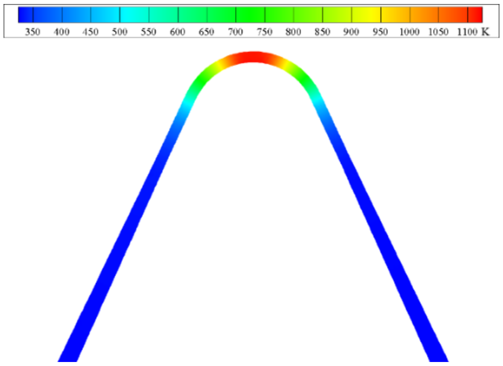

Supplementary Figure 4: Temperature profile along the heater from ANSYS simulation.

Next, we added a PEMF with diameter 3 μm and set a 20 μm gap between the heater and the fiber. The thermal conductivity of the PEMF was modeled using gel spun microfibers with k = 20 W m^{-1} K^{-1} [4]. We used emissivity value of the PEMF as 0.2[10]. The k of air was used as a constant value of 0.02 W m^{-1} K^{-1}. The temperature of the fiber reached 450 K (~180 °C). The temperature profile along the fiber is shown in Supplementary Fig. 5.

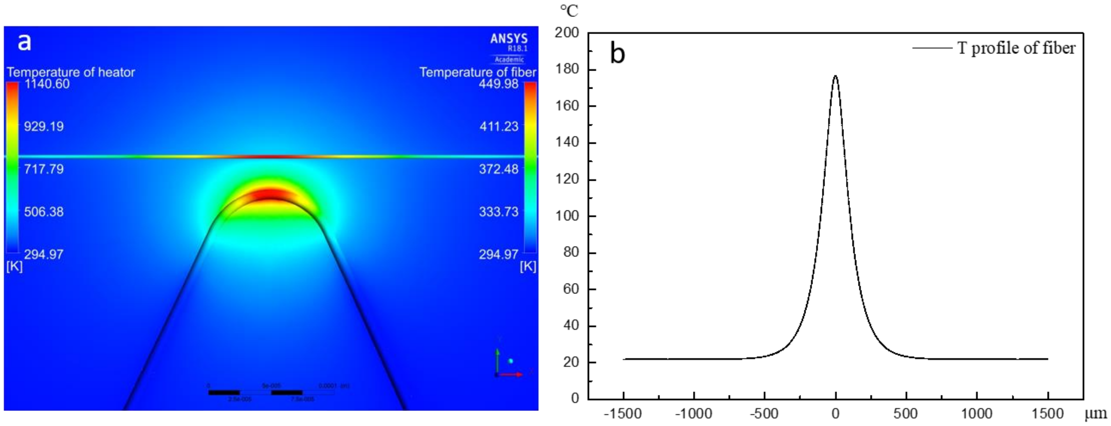

Supplementary Figure 5: Temperature profile of heater and a PEMF from ANSYS/FLUENT simulation. **a**) ANSY/FLUENT simulation temperature map of heater and a PEMF. **b**) Temperature profile of the PEMF. The maximum temperature of the fiber reaches 450 K (~180 °C).

Estimation of strain rate during local drawing

The molecular alignment within the fiber is correlated with increasing strain rate. We estimate that the strain rate in local drawing reaches up to 1400 s^{-1} compared to ~1000 s^{-1} in electrospinning[2] and ~1 s^{-1} in gel spinning[11]. Strain rate is defined as $\Delta l/(L_1\Delta t)$ where Δl is the extension of the fiber segment of length L_1 within Δt seconds.

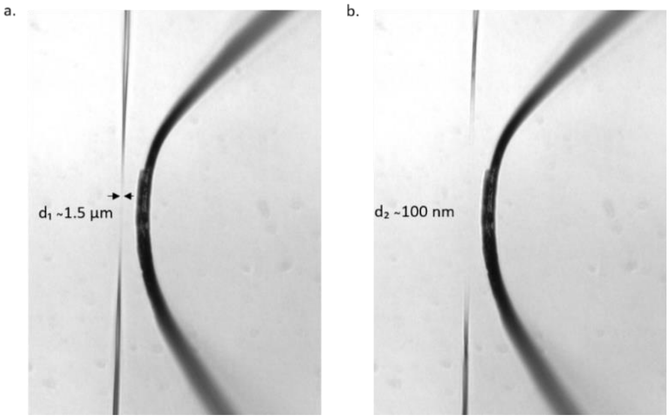

Supplementary Figure 6: PEMF undergoing high strain rate elongation. PENF (**a**) before and (**b**) after undergoing high strain rate elongation (diameter measured post measurement using Scanning electron microscopy (SEM)).

To estimate the strain rate during the local drawing, we used two consecutive image frames, as shown in Supplementary Fig. 6, from local drawing Supplementary Movie 1 submitted with the manuscript. Assuming volume conservation and a cylindrical fiber, $\pi d_1^2 L_1/4 = \pi d_2^2 L_1/4$ where d_1 is the initial diameter, d_2 is the final diameter after the local drawing, and $L_2$is the final length of the fiber. Assuming, $\Delta l \gg L_1$, we can show that ${L_2}/{L_1} = {(L_2 + \Delta l)}/{L_1} \approx {(\Delta l)}/{L_1} \approx {d_1^2}/{d_2^2}$. The fiber starts thinning at lower strain rate from 3 μm to around 1.5 μm before undergoing peak strain rate drawing. The video was obtained at an average frame rate of 6.3 frames per second. Therefore, the strain rate was estimated to be $(1.5\ \mu m)^2/((100\ nm)^2\ \times 0.16\ \mathrm{s}) \approx 1400\ \mathrm{s}^{-1}$.

Positioning PENF on the MEMS devices

As the fiber diameter decreases, it becomes more challenging to precisely locate and position it onto the MEMS test platforms. Electron microscopes cannot be used because of the sensitivity of polymer samples to electron beam radiation[12,13], which impairs the mechanical and thermal property enhancement[3,14]. Therefore, the manipulation of the PENF is limited to optical methods. The sample must also be properly aligned. For example, during tensile testing an unwanted bending moment can cause the loadcell to rotate and the fiber force will be underestimated. The miniscule sample size, the limited resolution of the optical microscope and unpredictable forces such as van der Waals, triboelectric and capillary forces due to moisture are factors that make it challenging to successfully place nanofibers. Instead of manipulating the PENF directly, the sample collector with a mounted PENF is maneuvered as shown Supplementary Fig. 7. The microfiber in the undrawn section is used as a reference to align the nanofiber. After aligning the PENF, the sample was cut by local melting a suitable distance away from the region tested using a micro heater.

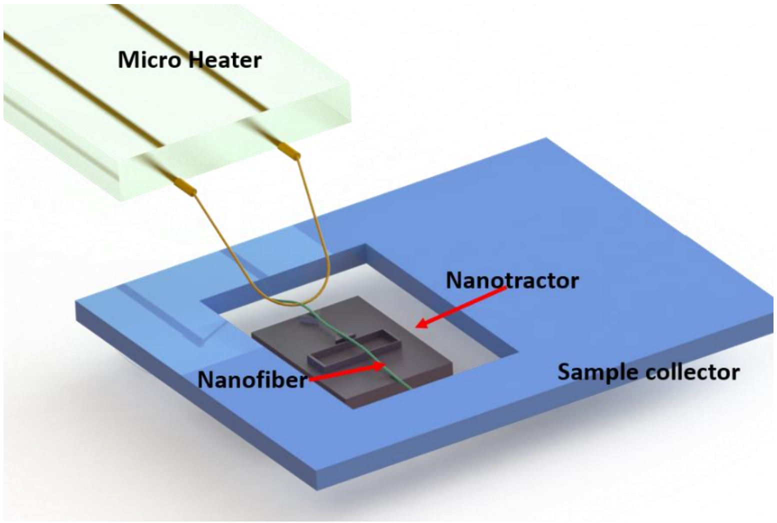

Supplementary Figure 7: Mounting of a nanofiber onto the Nanotractor using the sample collector. This is performed under an optical microscope using a 20X objective.

Supplementary Note 3: Characterization of PE powder, PEMF and PENF

SAED analysis of PENF

During the high strain rate fabrication step, PE crystals can undergo a phase transformation from the orthorhombic to a monoclinic phase[15]. Most SAED images indicated only the orthorhombic phase. One SAED pattern, Supplementary Fig. 8, reveals the monoclinic phase, in the orthorhombic matrix. We did not evaluate exact weight fraction of monoclinic phase, however, we expect it to be small. It has been measured for a highly drawn PE microfiber using Wide angle X-Ray scattering (WAXS) to be 4.3 %[16].

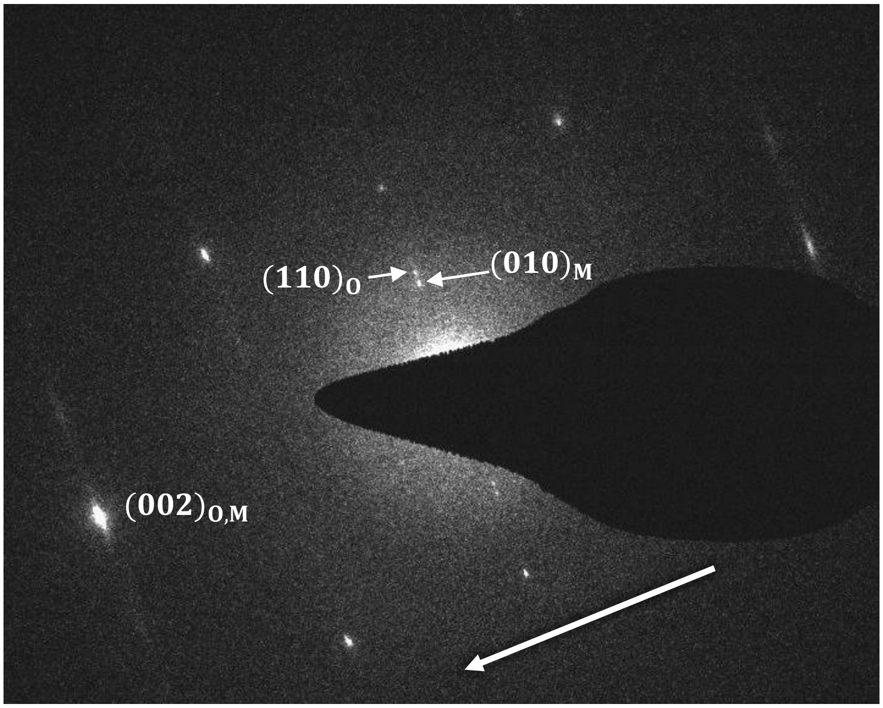

Supplementary Figure 8: SAED pattern of a PENF where a monoclinic phase is seen in addition to orthorhombic phase. Plane with subscript 'O' corresponds to the plane from orthorhombic structure whereas 'M' corresponds to the plane from monoclinic structure. Arrow indicates the drawing direction.

PE powder characterization using DSC

The PE powder (Sigma Aldrich, $M_w = 3 – 6 \times 10^6$ g mol^{-1}) used as the control was characterized using differential scanning calorimetry (DSC) to understand its initial crystallinity, as seen in Supplementary Fig. 9.

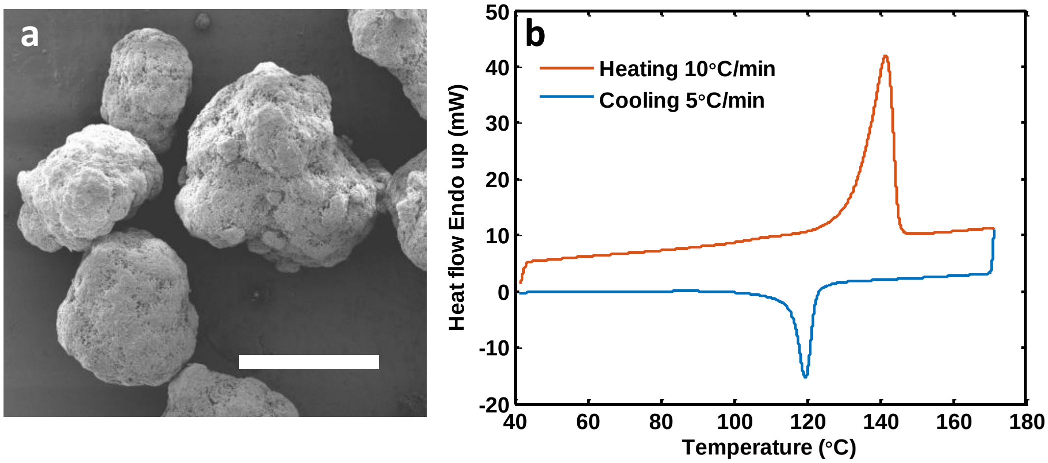

Supplementary Figure 9: a) SEM micrograph of PE powder particles. **b**) DSC curves of heating and cooling of PE powder. The melting point of PE powder is 141 °C. The enthalpy area of melting endotherm from 105 °C to 155 °C is found to be 1743.8 mJ. Scale bar, 100 μm.

A melting endotherm from the DSC can be used to measure the enthalpy of fusion of the polymer. The enthalpy of fusion of the sample can be compared with the theoretical crystal enthalpy of fusion to obtain the crystallinity of the sample as shown below[17].

$$\% \, \chi_{\mathrm{c}} = \frac{\Delta H_{\mathrm{s}}}{\Delta H_{\mathrm{f}}} \times 100 \tag{1}$$

Here χ_{c} is the % crystallinity of the sample, ΔH_{s} is the enthalpy of fusion of the sample and ΔH_{f} is the enthalpy of fusion of pure crystal 289.3 J g^{-1}. The enthalpy of fusion of PE powder was obtained to be 157.1 J g^{-1}. Therefore, the crystallinity is 54.3 %.

Structural damage of PENF during Raman characterization

Supplementary Fig. 10 shows Raman spectra of a PENF obtained at the same spot. They essentially overlap (within 5 %), suggesting that the fiber undergoes minimal structural damage during the characterization.

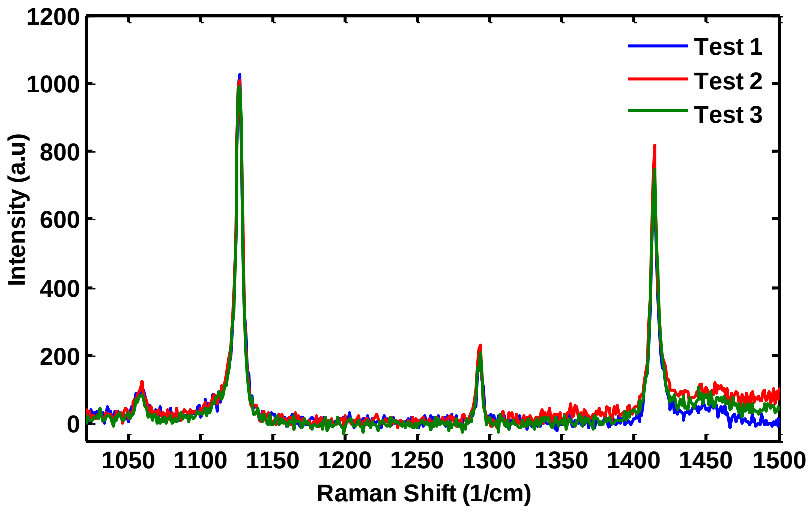

Supplementary Figure 10: Raman spectra of a PENF obtained at the same spot.

PE powder crystallinity characterization from micro-Raman

Following the method of Strobl and Hagedorn[18], the crystallinity of unoriented PE powder can be determined using micro Raman as follows:

$$\%\chi_c = \frac{A_{1415}}{A_{(1295+1305)}} \times \frac{100}{0.45} \quad (2)$$

where A_{1415} is the integral area under 1415 cm^{-1} Raman band and $A_{(1295+1305)}$ is the integral area of 1295 cm^{-1} and 1305 cm^{-1} Raman bands, which act as an internal standard. A spectrum is shown in Supplementary Fig. 11.

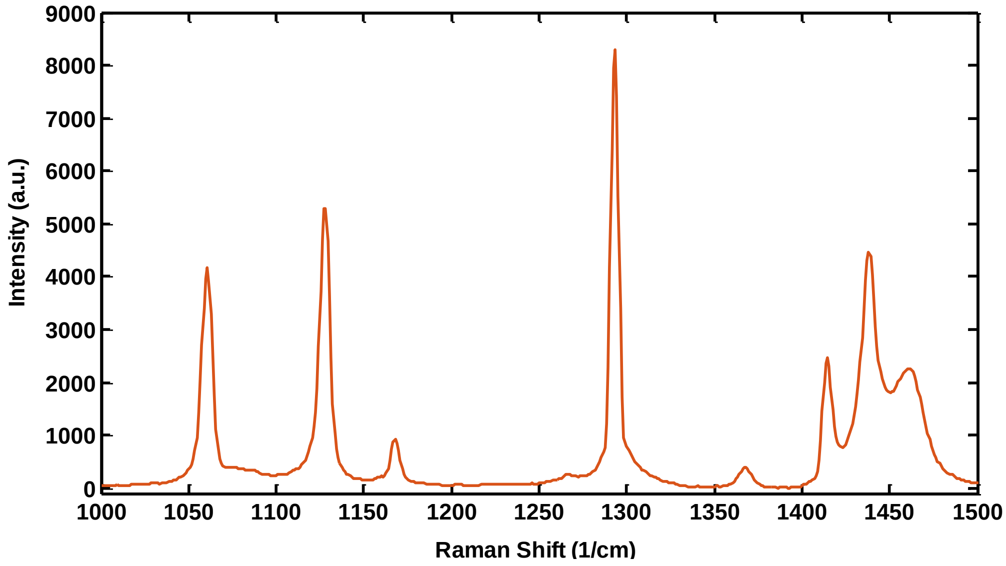

Supplementary Figure 11: Micro Raman measurement of PE powder.

The Raman spectrum of PE powder was deconvoluted and the integral area under the 1295 cm^{-1}, 1305 cm^{-1} and 1415 cm^{-1} Raman bands were measured using curve fitting with Renishaw's Windows based Raman environment (WiRE) software (WiRE™/ Renishaw). Three separate Raman spectrum measurements of PE powder are tabulated in Supplementary Table 1 below:

Supplementary Table 1: Crystallinity of a PE powder particle from Raman measurements

	A_{1415}	$A_{(1295+1305)}$	% χ_c
1	16435.2	58746.2	**54.6**
2	16843.8	57660.6	**56.9**
3	17493.8	58383.1	**59.8**

An average crystallinity of 57.1 % was obtained from the analysis of Raman bands of PE powder, in good agreement with the crystallinity obtained from the DSC analysis. The small difference of 3% could be because the DSC measurement is an average of bulk while micro Raman measurement is for a local area of a powder particle.

Supplementary Note 4: Noise, uncertainty and contact resistance in thermal measurement

Noise equivalent thermal conductance of platinum resistance thermometer (PRT) micro devices

The thermal conductance assessment is limited by the ability to measure the temperature rise in the sensing island (denoted by subscript s) accurately. Following the sensitivity analysis of Shi *et al.*[19], the noise-equivalent thermal conductance (NEG_{s}) in the sensing island can be shown as

$$NEG_{\mathrm{s}} = G_{\mathrm{b}} \frac{NET_{\mathrm{s}}}{(\Delta T_{\mathrm{h}} - \Delta T_{\mathrm{s}})} \tag{4}$$

where G_{b} is the total thermal conductance (W K^{-1}) of the supporting SiN_x beams of an island, ΔT_{h} is the temperature rise in the heating island, ΔT_{s} is the temperature rise in the sensing island and NET_{s} is the noise-equivalent temperature (K) of the sensing island. Also,

$$NET_{\mathrm{s}} = \frac{NER_{\mathrm{s}}/R_{\mathrm{s}}}{TCR} \tag{5}$$

where NER_s is the noise-equivalent resistance (Ω) of the sensing island. Through measurement of a precision 1 MΩ resistor, $NER_{\mathrm{s}}/R_{\mathrm{s}}$ was found to be ~7.5×10^{-5}. The temperature coefficient of resistance (TCR) of the PRT was found to be ~2.6×10^{-3} K^{-1} at 150 K and ~2.1×10^{-3} K^{-1} at 300 K. Therefore, NET_{s} is ~29 mK at 150 K and ~36 mK at 300 K. The temperature fluctuation in the cryostat is ~10 mK after waiting for the global temperature to stabilize for an hour at temperatures above 150 K.

The thermal conductance of the suspended PRT beams, G_{b}, is 70 – 90 nW K^{-1} at 150 K and 90 – 110 nW K^{-1} at 300 K. The temperature difference between heating and sensing islands ($\Delta T_{\mathrm{h}} - \Delta T_{\mathrm{s}}$) is kept within 5 K. The NEG_{s} of the measurement system can be now found from Supplementary Equation 4. At 300 K, the NEG_{s} is ~0.85 nW K^{-1} and at 150 K it is ~0.52 nW K^{-}

[1]. This is around 1 % of the of the measured conductance, hence noise is not an issue in the measurements.

Background thermal conductance

Because the thermal conduction experiment was carried out at a high vacuum level of 2×10^{-7} Torr and the temperature difference between the islands was limited to 5 K, we expect negligible heat transfer from residual gas and radiation. A background thermal conductance, G_{bkgd}, measurement of an empty micro thermal device in the same experimental conditions verifies that G_{bkgd} is within the NEG_{s} as shown in Supplementary Fig. 12.

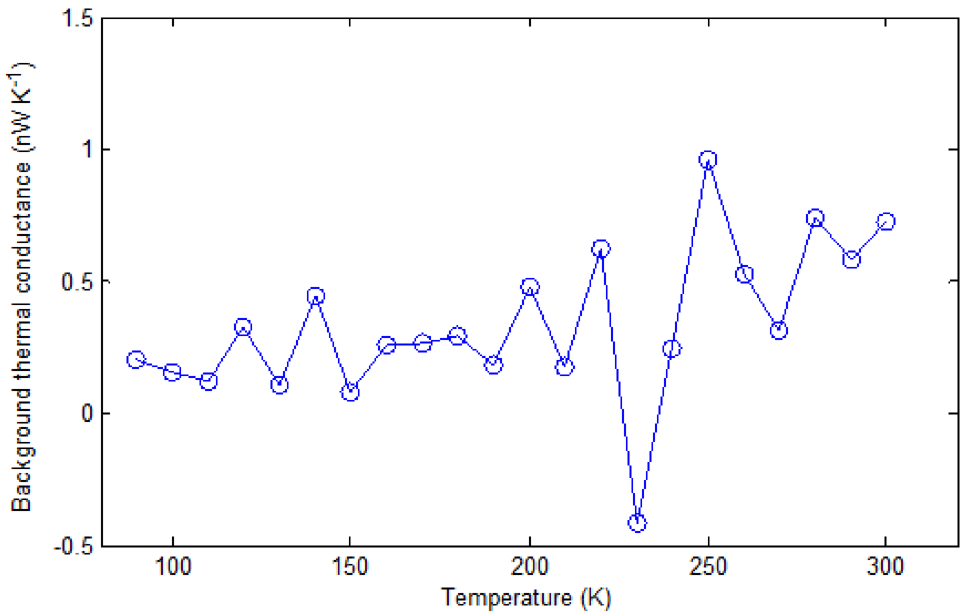

Supplementary Figure 12: Background thermal conductance of an empty device with gap 5 µm.

Uncertainty analysis in thermal conductivity

The thermal conductivity of an individual PENF is given by

$$k = \frac{GL}{A} = \frac{4GL}{\pi d^2} \tag{6}$$

where G is its thermal conductance and L and A are its length and cross-sectional area. Using the uncertainty propagation rule, the error in k can be expressed as

$$\left(\frac{\delta k}{k}\right)^2 = \left(\frac{\delta G}{G}\right)^2 + \left(\frac{\delta L}{L}\right)^2 + \left(2\frac{\delta d}{d}\right)^2 \tag{7}$$

where δ is the uncertainty. The values for $\left(\frac{\delta L}{L}\right)^2$ and $\left(2\frac{\delta d}{d}\right)^2$ are obtained from the SEM images as described later. From the analysis of Shi *et al.*[19,20],

$$G = \frac{Q}{(\Delta T_\mathrm{h} - \Delta T_\mathrm{s})}\left(\frac{\Delta T_\mathrm{s}}{\Delta T_\mathrm{h} + \Delta T_\mathrm{s}}\right) \tag{8}$$

where Q is the heat transferred to the heating island. The error in G can be written as

$$\left(\frac{\delta G}{G}\right)^2 = \left(\frac{\delta Q}{Q}\right)^2 + \left(\frac{\delta \Delta T_\mathrm{s}}{\Delta T_\mathrm{s}}\right)^2 + \left(\frac{\delta(\Delta T_\mathrm{h} - \Delta T_\mathrm{s})}{(\Delta T_\mathrm{h} - \Delta T_\mathrm{s})}\right)^2 + \left(\frac{\delta(\Delta T_\mathrm{h} + \Delta T_\mathrm{s})}{(\Delta T_\mathrm{h} + \Delta T_\mathrm{s})}\right)^2 . \tag{9}$$

When a dc current I is applied to the heating island, it can be shown that the heat transferred to the heating island is equivalent to

$$Q = I^2(R_\mathrm{h} + R_\mathrm{l}) = I^2\frac{(R_\mathrm{h} + R_\mathrm{l})}{R_\mathrm{h}}R_\mathrm{h} = I^2\gamma R_\mathrm{h} , \tag{10}$$

where R_h is the resistance of the heating island and R_l is the resistance of a current carrying SiN_x beam. The error in DC power in the heating island is given by,

$$\left(\frac{\delta Q}{Q}\right)^2 = \left(2\frac{\delta I}{I}\right)^2 + \left(\frac{\delta R_\mathrm{h}}{R_\mathrm{h}}\right)^2 \tag{11}$$

DC current up to 20 μA is applied with a Keithley 2400 source measurement unit with a high accuracy of $\pm$5 nA. Thus, the uncertainty (<0.07 %) due to heat input into the heating island (Q) is low.

The error in temperature rise in the heating or sensing island can be shown to be

$$\left(\frac{\delta \Delta T_\mathrm{i}}{\Delta T_\mathrm{i}}\right)^2 = \left(\frac{\delta \Delta R_\mathrm{i}}{\Delta R_\mathrm{i}}\right)^2 + \left(\frac{\delta \alpha_\mathrm{i}}{\alpha_\mathrm{i}}\right)^2 \tag{12}$$

where i = s (sensing) or h (heating), and $\alpha_{\mathrm{i}} \equiv {}^{dR_{\mathrm{i}}}/_{dT}$. A value for α_{i} is obtained from curve fitting of R_{i} and T where $\Delta T = 10$ K. The noise in R_{i} is small compared to T as $\left({}^{\delta R_{\mathrm{i}}}/_{R_{\mathrm{i}}}\right) \approx 7.5 \times 10^{-5}$. Then, $\delta(\Delta T) = \sqrt{2}\delta(T)$. Assuming the worst-case scenario, *NET* is added with the temperature fluctuation in the cryostat, $\delta(T) \approx 36\text{ mK} + 10\text{ mK} \approx 50\text{ mK}$. Then, $\delta(\Delta T) = \sqrt{2} \cdot 50\text{ mK} = 71\text{ mK}$. Hence, $\left({}^{\delta \alpha_{\mathrm{s}}}/_{\alpha_{\mathrm{s}}}\right) \approx {}^{71 \times 10^{-3}\text{ mK}}/_{10\text{ K}} = 0.71\ \%$. Zhu *et al.*[21] estimated $\left({}^{\delta \alpha_{\mathrm{i}}}/_{\alpha_{\mathrm{i}}}\right)$ from the least square linear fit of five R_{i} vs T measurements within 10 K for a 95 % confidence interval to be < 0.76 % ,which is consistent with our calculation.

The error in $\frac{\delta \Delta R_{\mathrm{i}}}{\Delta R_{\mathrm{i}}} \approx {}^{\sqrt{2}\delta(R_{\mathrm{i}})}/_{\Delta R_{\mathrm{i}}}$ can be estimated from the change in resistance during a heating ramp. At 100 K, ${}^{\delta \Delta R_{\mathrm{h}}}/_{\Delta R_{\mathrm{h}}} \approx {}^{\sqrt{2} \cdot 0.1275}/_{30} \approx 0.56\ \%$; ${}^{\delta \Delta R_{\mathrm{s}}}/_{\Delta R_{\mathrm{s}}} \approx {}^{\sqrt{2} \cdot 0.1275}/_{11} \approx 1.6\ \%$. Similarly, at 300 K, ${}^{\delta \Delta R_{\mathrm{h}}}/_{\Delta R_{\mathrm{h}}} \approx 0.6\ \%$; ${}^{\delta \Delta R_{\mathrm{s}}}/_{\Delta R_{\mathrm{s}}} \approx 2.15\ \%$.

Then, the error in ΔT_{s} and ΔT_{h} can now be estimated. At 100 K,

$$\left(\frac{\delta(\Delta T_{\mathrm{h}})}{\Delta T_{\mathrm{h}}}\right) = \sqrt{(0.56 \times 10^{-2})^2 + (0.71 \times 10^{-2})^2} \approx 0.90\ \%$$

$$\left(\frac{\delta(\Delta T_{\mathrm{s}})}{\Delta T_{\mathrm{s}}}\right) = \sqrt{(1.6 \times 10^{-2})^2 + (0.71 \times 10^{-2})^2} \approx 1.75\ \%$$

Similarly, at 300 K,

$$\left(\frac{\delta(\Delta T_{\mathrm{h}})}{\Delta T_{\mathrm{h}}}\right) = \sqrt{(0.6 \times 10^{-2})^2 + (0.71 \times 10^{-2})^2} \approx 0.93\ \%$$

$$\left(\frac{\delta(\Delta T_{\mathrm{s}})}{\Delta T_{\mathrm{s}}}\right) = \sqrt{(2.15 \times 10^{-2})^2 + (0.71 \times 10^{-2})^2} \approx 2.26\ \%$$

Now, using $a_{\mathrm{h}} = \left(\frac{\delta(\Delta T_{\mathrm{h}})}{\Delta T_{\mathrm{h}}}\right)$ $and\ a_s = \left(\frac{\delta(\Delta T_{\mathrm{s}})}{\Delta T_{\mathrm{s}}}\right)$

$$\left(\frac{\delta(\Delta T_{\mathrm{h}}-\Delta T_{\mathrm{s}})}{(\Delta T_{\mathrm{h}}-\Delta T_{\mathrm{s}})}\right)=\frac{\sqrt{[\delta(\Delta T_{\mathrm{h}})]^2+[\delta(\Delta T_{\mathrm{s}})]^2}}{(\Delta T_{\mathrm{h}}-\Delta T_{\mathrm{s}})}=\frac{\sqrt{(a_{\mathrm{h}}.\Delta T_{\mathrm{h}})^2+(a_{\mathrm{s}}.\Delta T_{\mathrm{s}})^2}}{(\Delta T_{\mathrm{h}}-\Delta T_{\mathrm{s}})}$$

Similarly,

$$\left(\frac{\delta(\Delta T_{\mathrm{h}}+\Delta T_{\mathrm{s}})}{(\Delta T_{\mathrm{h}}+\Delta T_{\mathrm{s}})}\right)=\frac{\sqrt{[\delta(\Delta T_{\mathrm{h}})]^2+[\delta(\Delta T_{\mathrm{s}})]^2}}{(\Delta T_{\mathrm{h}}+\Delta T_{\mathrm{s}})}=\frac{\sqrt{(a_{\mathrm{h}}.\Delta T_{\mathrm{h}})^2+(a_{\mathrm{s}}.\Delta T_{\mathrm{s}})^2}}{(\Delta T_{\mathrm{h}}+\Delta T_{\mathrm{s}})}$$

To estimate the error of above two expressions, let us consider the typical case of PE nanofibers at 100 K and 300 K. At 100K, we observe that for $\Delta T_{\mathrm{h}} \approx 4.5\,K$, $\Delta T_{\mathrm{s}} \approx 2.0\,K$. Thus, $\Delta T_{\mathrm{h}}+\Delta T_{\mathrm{s}} \approx 6.5\,K$ and $\Delta T_{\mathrm{h}}-\Delta T_{\mathrm{s}} \approx 2.5\,K$

$$\left(\frac{\delta(\Delta T_{\mathrm{h}}-\Delta T_{\mathrm{s}})}{(\Delta T_{\mathrm{h}}-\Delta T_{\mathrm{s}})}\right)=\frac{\sqrt{(0.9\times10^{-2}\times4.5)^2+(1.75\times10^{-2}\times2.0)^2}}{(2.5)}=2.14\,\%$$

Similarly,

$$\left(\frac{\delta(\Delta T_{\mathrm{h}}+\Delta T_{\mathrm{s}})}{(\Delta T_{\mathrm{h}}+\Delta T_{\mathrm{s}})}\right)=\frac{\sqrt{(0.9\times10^{-2}\times4.5)^2+(1.75\times10^{-2}\times2.0)^2}}{(6.5)}=0.82\,\%$$

The total error in thermal conductance is then, using Supplementary Equation 10,

$$\left(\frac{\delta G}{G}\right)=\sqrt{(0.07\times10^{-2})^2+(1.75\times10^{-2})^2+(2.14\times10^{-2})^2+(0.82\times10^{-2})^2}=2.88\,\%$$

At 300 K, we observe that for $\Delta T_{\mathrm{h}} \approx 5\,K$, $\Delta T_{\mathrm{s}} \approx 1.4\,K$. Thus, $\Delta T_{\mathrm{h}}+\Delta T_{\mathrm{s}} \approx 6.4\,K$ and $\Delta T_{\mathrm{h}}-\Delta T_{\mathrm{s}} \approx 3.6\,K$. The total error in thermal conductance is then, using Supplementary Equation 10,

$$\left(\frac{\delta G}{G}\right)=\sqrt{(0.07\times10^{-2})^2+(2.26\times10^{-2})^2+(1.71\times10^{-2})^2+(0.96\times10^{-2})^2}=2.99\,\%$$

This error is for a single measurement of $G=\frac{Q}{(\Delta T_{\mathrm{h}}-\Delta T_{\mathrm{s}})}\left(\frac{\Delta T_{\mathrm{s}}}{\Delta T_{\mathrm{h}}+\Delta T_{\mathrm{s}}}\right)$, where heat flux ($Q$) is determined by an applied dc current I. In our measurement, we collect 250 data points for current from 10 – 20 μA to get the sample conductance more accurately.

For a typical sample with diameter of 100 nm and length 8 μm, the standard deviation in the diameter is found to be around 4 % and negligible in length. Then, the total error in the thermal conductivity at 300 K is

$$\left(\frac{\delta k}{k}\right) = \sqrt{(2.99 \times 10^{-2})^2 + (2 \times 4 \times 10^{-2})^2} = 8.54\ \%.$$

Thermal contact resistance

The thermal conductance measurement system uses two-probes and includes thermal contact resistance. Thermal contact resistance between the sample and the islands can result in an underestimation of the sample's k. The total thermal resistance of the system at steady state is the sum of thermal contact resistance between the heating island and the nanofiber ($R_{c,hi}$), the intrinsic thermal resistance of nanofiber (R_{s}) and the thermal contact resistance between the sensing island and the nanofiber ($R_{c,si}$). Therefore, the total thermal resistance ($R_{\mathrm{tot}} = 1/G_{\mathrm{s}}$) can be written as

$$R_{\mathrm{tot}} = R_{\mathrm{s}} + R_{\mathrm{c,hi}} + R_{\mathrm{c,si}} \tag{13}$$

The heating (h) and sensing (s) sides have the same geometrical properties and same contact mechanism, so we assume that $R_{\mathrm{c,hi}} \approx R_{\mathrm{c,si}} \approx R_{\mathrm{c}}$. Then

$$R_{\mathrm{tot}} = R_{\mathrm{s}} + 2R_{\mathrm{c}} \tag{14}$$

To measure the sample's k accurately, $2R_c$ should be negligible compared to sample's intrinsic thermal resistance ($R_{\mathrm{s}} = 4L_{\mathrm{s}}/\pi d_{\mathrm{s}}^2 k_{\mathrm{s}}$). To achieve $2R_{\mathrm{c}} \ll R_{\mathrm{s}}$, either R_{s} can be increased or $2R_{\mathrm{c}}$ can be lowered. For high k samples, such as PENF, intrinsic thermal resistance (R_{s}) can be increased by making the nanofiber long and the cross-section area small. However, this will also reduce the heat flux significantly. Consequently, the temperature rise of sensing side will be lessened, making accurate measurement difficult. Alternatively, thermal contact resistance can

be decreased to make $2R_c \ll R_s$. A platinum or graphite coating, using electron beam or focused ion beam (FIB), has been extensively used in literature to reduce $2R_c$[22,23]. However, high-energy electron/ion beam amorphized our sample and reduced the thermal conductivity enhancement to bulk as shown in Supplementary Fig. 13.

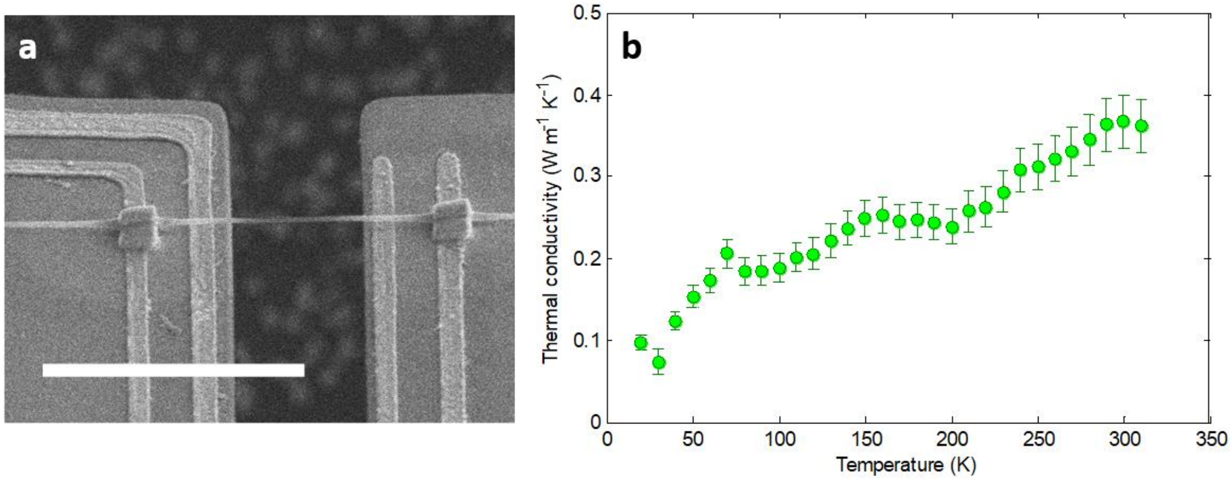

Supplementary Figure 13: a) Platinum coating using FIB on PENF. **b**) k of PENF with platinum contact. The k values are similar to that of bulk PE. Scalebar, 10 μm.

Due to sensitivity of our samples to electron/ion beam, we used capillary-assisted adhesion to minimize $2R_c$. In this process an isopropanol (IPA) drop is first placed on top of the thermal device. The evaporation of IPA from the device is closely monitored under an optical microscope. After the liquid meniscus connecting the islands breaks, but before the liquid layer from the suspended islands evaporates, a pre-aligned nanofiber is brought into contact with the islands. The liquid wets the fiber, and the evaporating liquid surface pulls the fiber down to the pads due to surface tension. However, $2R_c$ remains significant comparable to R_s. Following the analysis of Yu *et al*., the total $2R_c$ is given as[22]

$$2R_{\mathrm{c}} = \frac{2}{\sqrt{\frac{k_{||}A_{\mathrm{cr}}}{R'_{\mathrm{c}}} tanh\left(l_{\mathrm{c}} \sqrt{\frac{1}{k_{||}A_{\mathrm{cr}}R'_{\mathrm{c}}}} \right)}} \quad (15)$$

where $k_{||}$ is axial thermal conductivity of the nanofiber sample, A_{cr} is the cross – sectional area, R'_c is the constriction resistance per unit axial length in contact and l_c is the total length of nanofiber in contact with an island. R'_c is calculated using the line contact model of McGee *et al.* for a cylinder on a flat substrate[24,25]

$$R'_{\mathrm{c}} = \frac{1}{\pi k_{\perp}} ln\left(\frac{4d}{w}\right) - \left(\frac{1}{2k_{\perp}}\right) + \frac{1}{\pi k_{\mathrm{pt}}} ln\left(\frac{2d}{\pi w}\right) \quad (16)$$

where $k_{\perp}$ (0.33 W m^{-1} K^{-1} [26]) is the radial thermal conductivity of the nanofiber sample, *d* is the diameter of the sample, *w* is the contact width of nanofiber on the substrate and k_{pt} (70 W m^{-1} K^{-1}) is the thermal conductivity of platinum.

Contact width estimation

We need to know the contact width (w) between a nanofiber sample and an island to calculate $2R_{\mathrm{c}}$ using Supplementary Equations 15 and 16. Elastic plane strain analysis can be applied to obtain *w* assuming the van der Waals force represents the applied force[24,25].

For an elastic cylinder on a flat substrate, Bahadur *et al.*[25] derived *w* due to elastic deformation from the van der Waals force given as:

$$w = \sqrt{\left(\frac{16F_{\mathrm{vdw}}E_{\mathrm{m}}d}{\pi}\right)} \quad (17)$$

where F_{vdw} is the van der Waals force per unit contact length between the nanofiber and the island, and E_{m} is the effective modulus defined as

$$E_{\mathrm{m}} = \frac{1}{2}\left(\frac{1-\nu_{\mathrm{s}}^2}{E_{\mathrm{s}}} + \frac{1-\nu_{\mathrm{Pt}}^2}{E_{\mathrm{Pt}}}\right) \tag{18}$$

where $\nu_{\mathrm{s}} = 0.4$[27] is the Poisson's ratio of PE in cross plane-axial direction, $E_{\mathrm{s}} \approx 3.5$ GPa[26] is the elastic modulus of the highly drawn PE in cross plane direction, $\nu_{\mathrm{Pt}} = 0.36$ [28] is the Poisson's ratio of thin Pt film and $E_{\mathrm{Pt}} \approx 140$ GPa [29] is the elastic modulus of nanoscale Pt film.

The van der Waals force per unit contact length for a cylinder-flat substrate is given as[30]

$$F_{\mathrm{vdw}} = \frac{A_{\mathrm{PE-Pt}}\sqrt{d}}{16h^{5/2}} \tag{19}$$

where $A_{\mathrm{PE-Pt}}$ is a Hamaker constant for a PE-Pt interface and h is the separation distance between the PE nanofiber and Pt estimated as the sum of van der Waal's radii of Pt and C[31]. The Hamaker constant for a PE-Pt interface is

$$A_{\mathrm{PE-Pt}} = \sqrt{A_{\mathrm{PE-PE}}A_{\mathrm{Pt-Pt}}} \tag{20}$$

Hamaker constant value for a PE-PE interface is 8.43×10^{-20} J at room temperature[32]. Similarly, we used Hamaker constant for Pt-Pt interface (20×10^{-20} J) interacting in water at room temperature[33]. $A_{\mathrm{PE-Pt}}$ was estimated to be 13×10^{-20} J.

Higher $k_{||}$ of the sample leads to a lower $2R_c$ (per Supplementary Equation 15) as shown in Supplementary Fig. 14. We chose upper (100 W m^{-1} K^{-1}) and lower bound (50 W m^{-1} K^{-1}) from previously reported $k_{||}$ values at room temperature for an ultra-drawn PE nanofiber[34] to estimate the range of $2R_{\mathrm{c}}$ of our sample.

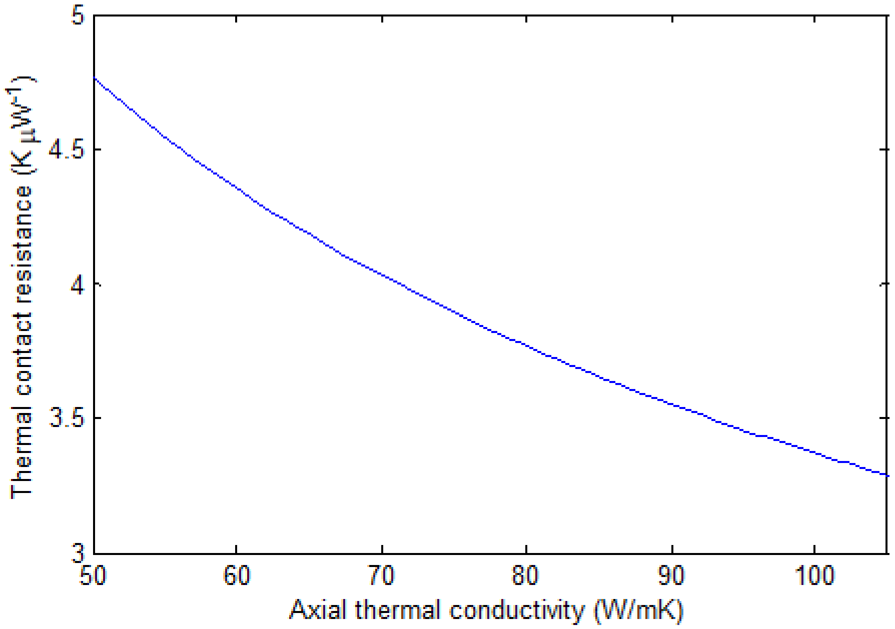

Supplementary Figure 14: Thermal contact resistance ($2R_c$) as a function of axial thermal conductivity of a PENF.

For a typical 100 nm PE sample, the w was found to be approximately 8.3 nm resulting in $2R_c \approx 3.31 \times 10^6 - 4.63 \times 10^6$ K W^{-1}. This is, 10 – 30 % of R_{tot}, as can be seen by applying $R_s = 4L_s/(\pi d_s^2 k_s)$ to the data in main text Fig. 3e.

Supplementary Note 5: Uncertainty in tensile strength measurement

Load cell stiffness estimation of the Nanotractor Platform

The stiffness of the loadcell, as shown in Fig. 5a of the main text, was estimated from the dimensions of the loadcell beams measured from the SEM images. Four identical fixed-guided beams determine its stiffness. The load cell was designed such that it was stiff enough to exert sufficient force to test the sample until failure and compliant enough to have good force resolution. The load cell beam stiffness is given by $k_L = Ehw^3/L^3$ where E, h, w *and* L are Young's modulus, height, width and length of the beam respectively.

Error analysis in stress using the Nanotractor Platform

Tensile strength (σ_{ts}) is the maximum stress that the sample can withstand before failing. It can be expressed as

$$\sigma_{\mathrm{ts}} = \frac{F_{\max}}{A} \tag{21}$$

where $F_{\max}$ is the maximum force. This can be rewritten as

$$\sigma_{\mathrm{ts}} = \frac{4k\Delta x_{\mathrm{loadcell,max}}}{\pi d^2} \tag{22}$$

The total uncertainty in σ_{ts} is also estimated using the uncertainty propagation rule:

$$\left(\frac{\delta\sigma_{\mathrm{ts}}}{\sigma_{\mathrm{ts}}}\right)^2 = \left[\left(\frac{\delta k_{\mathrm{L}}}{k_{\mathrm{L}}}\right)^2 + \left(\frac{\delta(\Delta x_{\mathrm{loadcell}})}{\Delta x_{\mathrm{loadcell}}}\right)^2 + \left(2\frac{\delta d}{d}\right)^2\right] \tag{23}$$

The load cell stiffness k_{L} is given by

$$k_{\mathrm{L}} = \frac{Ehw^3}{L^3} \tag{24}$$

$$\left(\frac{\delta k_{\mathrm{L}}}{k_{\mathrm{L}}}\right)^2 = \left(\frac{\delta E}{E}\right)^2 + \left(\frac{\delta h}{h}\right)^2 + \left(3\frac{\delta w}{w}\right)^2 + \left(3\frac{\delta L}{L}\right)^2 \tag{25}$$

Sample S1 with d = 85 ± 4 nm was measured using a load cell with k_{L} = 5.4 N m^{-1}. The uncertainties in length and width of the loadcell beam were calculated from five measurements along the loadcell beam.

$$\left(\frac{\delta L}{L}\right) \sim 0.05\ \% \quad and \quad \left(\frac{\delta w}{w}\right) = 0.6\ \%$$

The error in Young's modulus of the polysilicon (164.3 ± 3.2 GPa) and beam height (2.25 µm, std 6.3 nm) has been measured on MEMS devices fabricated with SUMMiT V™ method[35,36].

$$\left(\frac{\delta h}{h}\right) = 0.28\ \% \ and \ \left(\frac{\delta E}{E}\right) = 2\ \%$$

Thus, total uncertainty in stiffness $\left(\frac{\delta k_{\mathrm{L}}}{k_{\mathrm{L}}}\right)$ of the k_{L} = 5.4 N m^{-1} loadcell is 2.7 %. The loadcell extension (12 μm) is significantly larger than pattern matching resolution (4 nm) so $\left(\frac{\delta(\Delta x_{\mathrm{loadcell}})}{\Delta x_{\mathrm{loadcell}}}\right) < 0.1$ %. The major source of error is the fiber diameter:

$$\left(\frac{\delta d}{d}\right) = 4.7\ \%$$

Using Supplementary Equation 23, the uncertainty (one sigma) in stress is, $\left(\frac{\delta \sigma_{\mathrm{ts}}}{\sigma_{\mathrm{ts}}}\right) = 9.8\ \%$

Supplementary Note 6: Gripping of PENF for tensile measurement

Gripping by mechanical locking using a dog-bone

The surface energy of PE is low (31 mJ m^{-2}) and the absence of a polar component (similar to PTFE 19 mJ m^{-2}) makes it difficult to wet with adhesives[37,38]. There are several commercial adhesives such as Loctite 3032, Loctite 3035, Scotch weld DP 8005, Scotch weld DP 8010, Loctite plastic bonding system, and TAP poly-weld adhesive that are designed for low surface energy olefins such as polyethylene and polypropylene. However, their viscosity, working life and/or requirement to surface treat the sample made them unsuitable for this work. All available glues were tried. None worked successfully.

Increasing the surface energy of PE is the most common way at the macroscale to enhance PE adhesion. The surface energy can be altered by flame discharge, corona discharge, acid treatment and plasma treatment[38]. These surface treatments are destructive which will alter the surface chemistry and property of the sample via oxidation. The length scale of the surface where these surface treatments affect the material is similar to the diameter of our sample[38]. Thus, these methods are not viable. Surface roughening is another method that has been frequently used at

the macroscale. The glues within the rough surface provide additional force due to mechanical locking. However, with our sample size mechanical roughening of the fiber is not possible. Adhesion of an epoxy matrix with PE can also be enhanced by mixing reactive graphitic carbon nanofibers (r-GNF)[39]. However, dispersion is always an issue when mixing r-GNF with glues. There have been previous measurements where an adhesive was used to fix the polyethylene nanofiber during an AFM based three-point bending test. This was successful because the applied force was three orders of magnitude smaller and tested for very small displacement [40].

Here, we introduce a mechanical locking method to increase the gripping force. During localized drawing of a PEMF, the melt forms elongated globules at the edges of the PENF, similar to a dog bone, in response to the combined surface tension and drawing forces. Placing the glue (Gorilla epoxy) between the dog bone and the PENF yields mechanical locking which provides a significant additional resisting force. Furthermore, the thicker section of the dog-bone (3 – 5 µm) makes it easy to locate and manipulate the sample under the optical microscope. A schematic of the dog bone within an epoxy is shown in Supplementary Fig. 15a. Supplementary Fig. 15c also shows local crazing of the glue as the dog bone is pulled into it, indicating successful mechanical locking. However, it also reveals that crazing is one slip mechanism. Supplementary Figs. 15 d and e show the taper at the tip of dog bone.

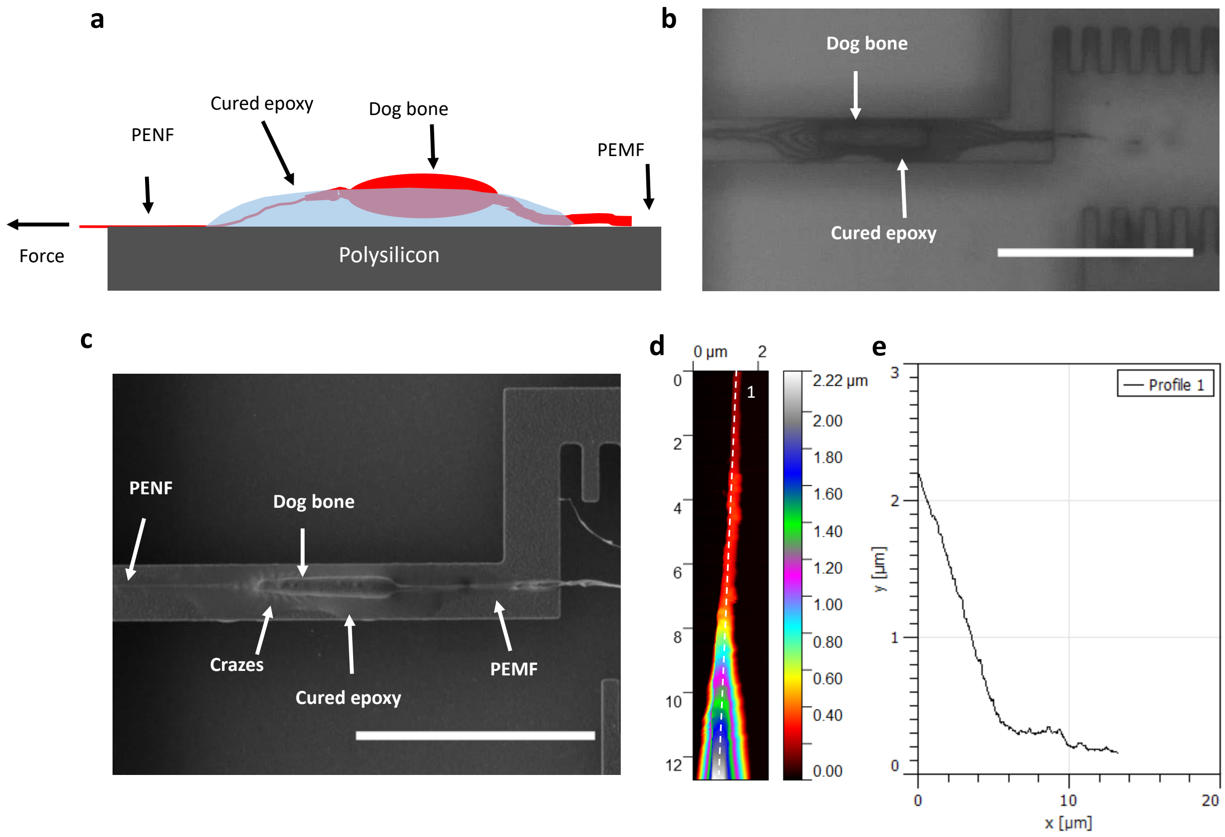

Supplementary Figure 15: PE nanofiber (PENF) on the left side of the dog bone for additional mechanical gripping, with PE microfiber (PEMF) on the right. **a**) Schematic of mechanical locking of the dog bone within cured epoxy. **b**) Optical image of the dog bone before testing. **c**) SEM image of the same dog bone after testing. The streaks at the left edge of the dog bone in **c** are crazes due to the deformation of the cured epoxy. They are only on the left side of the dog bone, indicating successful gripping of the test sample (the sample in Fig. 4 of the main text was not tested and does not exhibit crazing). **d**) Atomic force microscopy (AFM) image of tip of a dog bone (SEM image not shown here). **e**) Height profile of the dog bone in **d** labelled '1'. Scale bars, 25 µm (**b,c**).

Evaluation of glue adhesion and performance of dog bone

From composites theory[41], the maximum force (F_{max}) up to which a sample with a diameter d_s was tested before it slipped can be used to obtain the shear strength (σ_{shear}) of glue/PE, given as

$$\sigma_{\text{shear}} = \frac{F_{\max}}{\pi d_s l_{\text{glue}}} \quad (24)$$

Supplementary Equation 24 can be re-written as

$$\frac{F_{\max}}{\pi l_{\text{glue}}} = \sigma_{\text{shear}} d_s \quad (25)$$

where l_{glue} is the length of the nanofiber on which glue is applied. In Supplementary Fig. 16a, $F_{\max}/\pi l_{\text{glue}}$ is plotted against d_s. The slipped samples have a slope of 4.7 MPa which is the shear strength of glue with the PE fiber. This is on par with the shear strength of Loctite® AA 3032/ Henkel which is a specialized glue for PE (6 MPa with high density PE). Samples tested till failure experience a significantly higher force per unit length. This additional force per unit length required to test till failure is contributed by the mechanical locking of the sample.

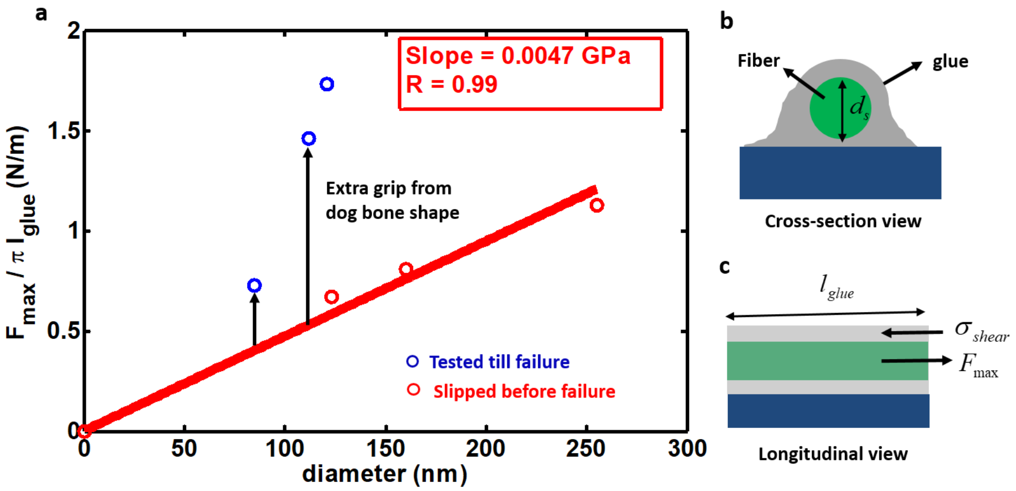

Supplementary figure 16: Estimation of glue shear strength. **a**) Shear force per unit glued length versus diameter of the fiber. The slope gives the shear modulus of glue with PE fiber. (**b)** and (**c)** are schematic cross section and longitudinal views of the fiber in the glue.

Now, we can also estimate the length of glued section required to hold a typical 100 nm PENF with tensile strength (σ_{ts}) of 10 GPa. It can be shown using Supplementary Equations 21 and 24 that,

$$l_{\mathrm{glue}} = \frac{\sigma_{\mathrm{ts}} d_{\mathrm{s}}}{4\sigma_{\mathrm{shear}}} \approx 53\ \mu m$$

This could not be achieved in the present experiment because the pad size is 30 μm.

Supplementary Note 7: Diameter characterization of PENF

Diameter measurement and electron beam swelling in PE

Both the strength and thermal conductivity measurements depend on the diameter of the PENF, so it is crucial to characterize its value accurately. However, swelling of the PENF due to electron beam irradiation during the SEM imaging makes the measurement challenging.

To characterize the SEM damage as well as morphology of the fiber, the diameter of an as-drawn fiber was first measured in an atomic force microscope (AFM) followed by SEM. Prior to SEM, the nanofiber was coated with 3 nm platinum to minimize charging during SEM. This does not significantly change the diameter of the nanofiber as the platinum deposition is not conformal.

AFM (Supplementary Figs. 17a and b) and SEM (Supplementary Figs. 17c and d) images show where the same three regions have been measured. A region with distinct patterns of debris on silicon wafer was chosen so it would be easy to find in both AFM and SEM. The SEM measured diameters are 10.7 %, 11.9 % and 11.9 % larger than those from AFM. Supplementary Fig. 17d also shows the morphological damage in PE nanofiber. The chains in the as-drawn fiber are scissioned by the irradiation. The observed ripples show the sheesh-kebab structure that evolves thereafter.

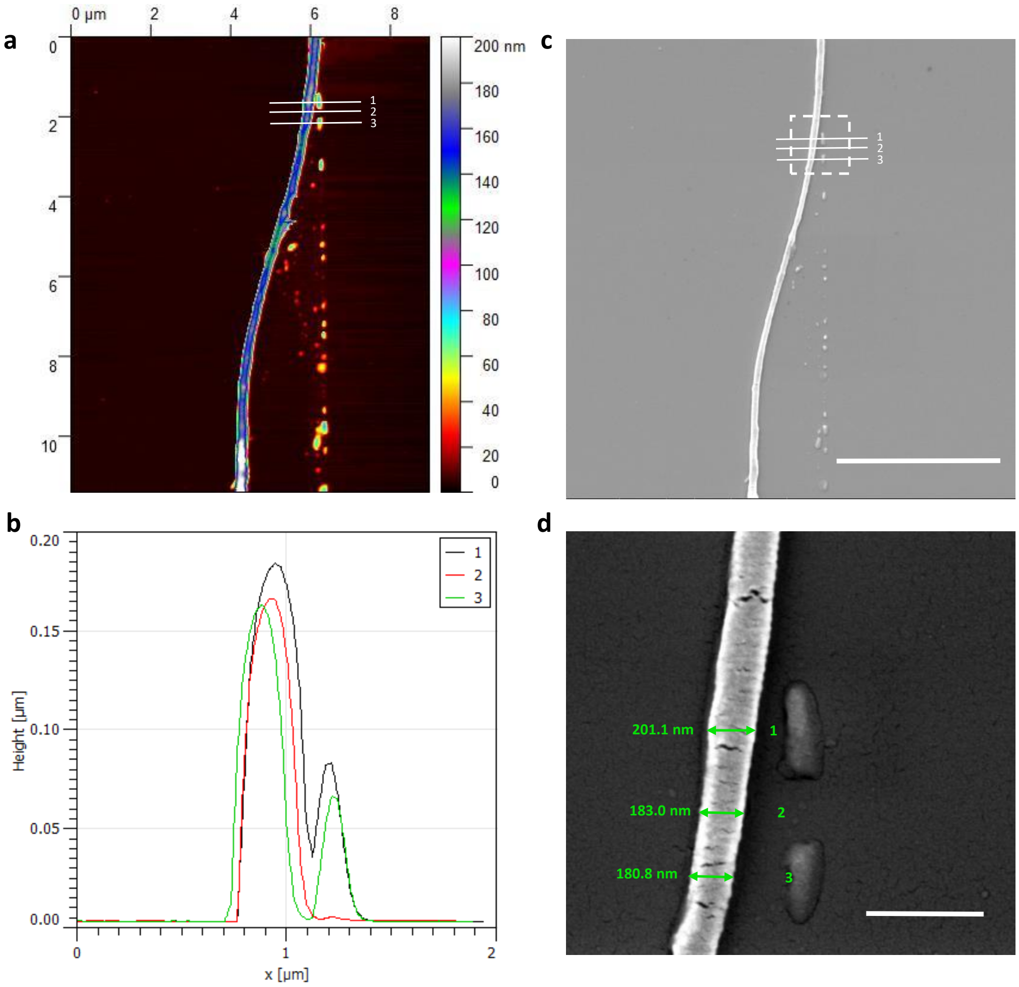

Supplementary Figure 17: Electron beam swelling in PENF. **a**) AFM image of a nanofiber **b**) Height profile of lines drawn in (**a**). Horizontal distance is larger because of the convolution of the AFM tip. **c**) SEM image of PENF **d**) Magnified view of inset of (**c**). Scale bars 5 μm (**c**) and 500 nm (**d**).

Supplementary Fig 18 shows the separation between the thermal measurement platforms due to increasing electron beam irradiation. The length of the fiber decreases in (b) relative to (a). The diameter of the swollen fiber is measured. Assuming that the volume does not change, one can obtain the initial diameter, d_{ini}, of the nanofiber using the formula $d_{\mathrm{ini}} = d_{\mathrm{swoln}}\sqrt{(length_{\mathrm{swoln}}/length_{\mathrm{ini}})}$.

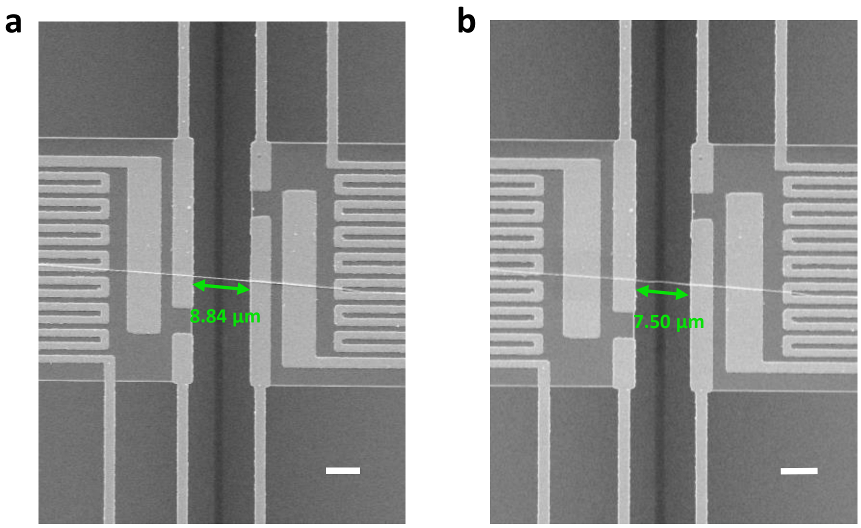

Supplementary Figure 18: Diameter measurement for k evaluation. **a**) SEM micrograph of the initial fiber **b**) SEM micrograph after SEM exposure. Scale bars, 5 µm.

Unlike thermal measurements, the PENF breaks during strength measurements. When imaged in SEM, it coils rapidly due to irradiation near the free end. In the literature, the diameter of polymer fibers has been measured from an untested section[14]. However, this is not feasible here because the fiber exhibits tapering. Hence, the diameter is measured within the test section by SEM. As discussed in the manuscript, the fiber fails by ductile failure (low k_L) or by extreme necking (high k_L). In the first case, the fiber diameter was measured (10 µm) away from the fractured end as shown in Supplementary Fig 19a. In a crystalline PEMF, the failure strain was 6.5 % at a strain rate comparable to this work (10^{-3} s^{-1})[42]. So, assuming constant volume deformation, the diameter of the fiber decreases by 3.2%. However, the controlled experiment (Supplementary Fig 17) showed that the measured diameter of the fiber was overestimated by 12 % due to irradiation. The value used to calculate strength is from SEM without correction, and therefore the reported strength is a lower bound.

In case of extreme necking, the local region can undergo large non-uniform deformation. Therefore, the diameter in this case was measured close to the clamped region as shown in Supplementary Fig 19c.

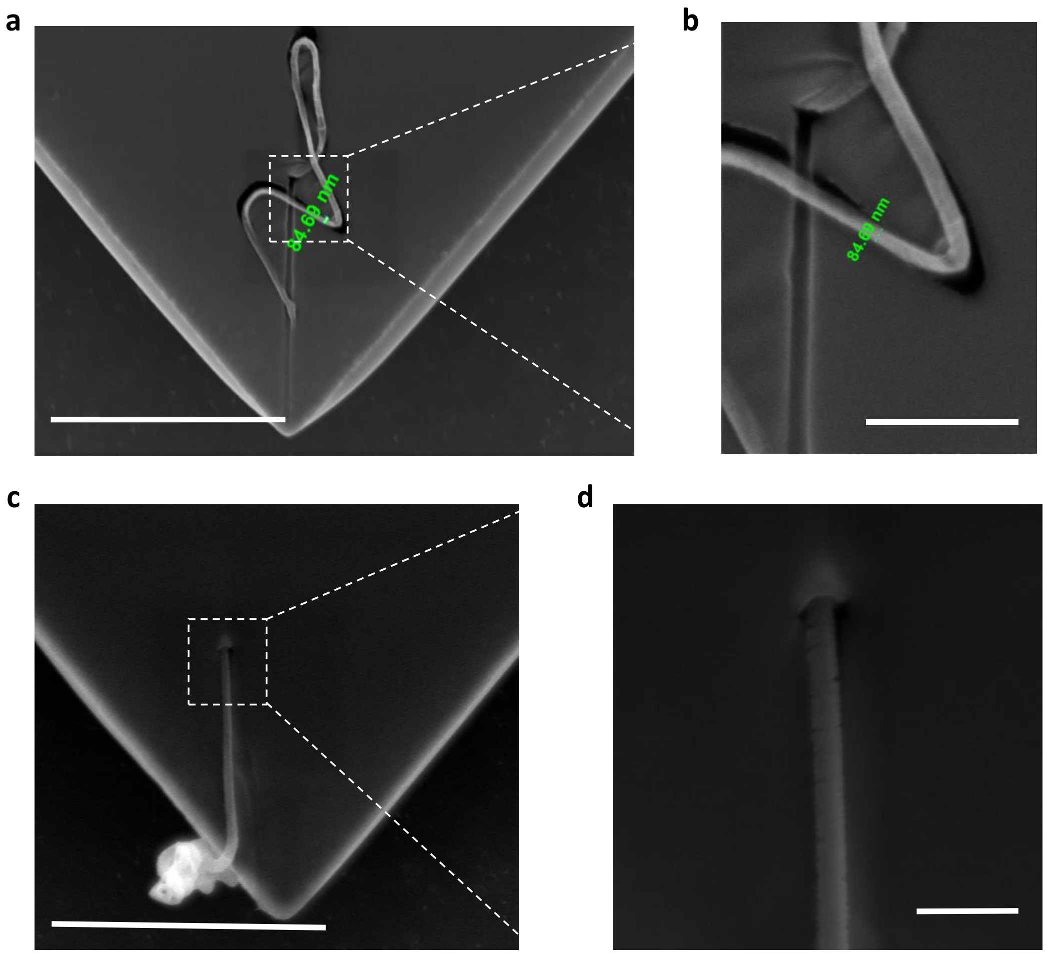

Supplementary Figure 19: Diameter measurement for σ_{ts} evaluation. **a**) Fractured sample. **b**) Zoom in of inset of a. **c**) Extremely necked sample. The diameter is measured far away from necked region. The coiling is prominent in freely suspended sample compared to the sample on the substrate. Scale bars, 5 µm (**a**,**c**), 1 µm (**b**), 500 nm (**d**).

Extreme necking

As stated in the manuscript, the samples tested with the high stiffness loadcell experienced extreme necking. Under electron beam irradiation, the free end of the PENF coils rapidly. Therefore, a measurement was performed using AFM on an extremely necked region of a mechanically probed sample. The AFM height values of 7, 1.4 and 1.2 nm in Supplementary Fig. 20b are obtained from regions 1, 2 and 3 in Supplementary Fig. 20a. This shows that the fibers deform by extreme necking rather than a typical local fracture with a short neck region.

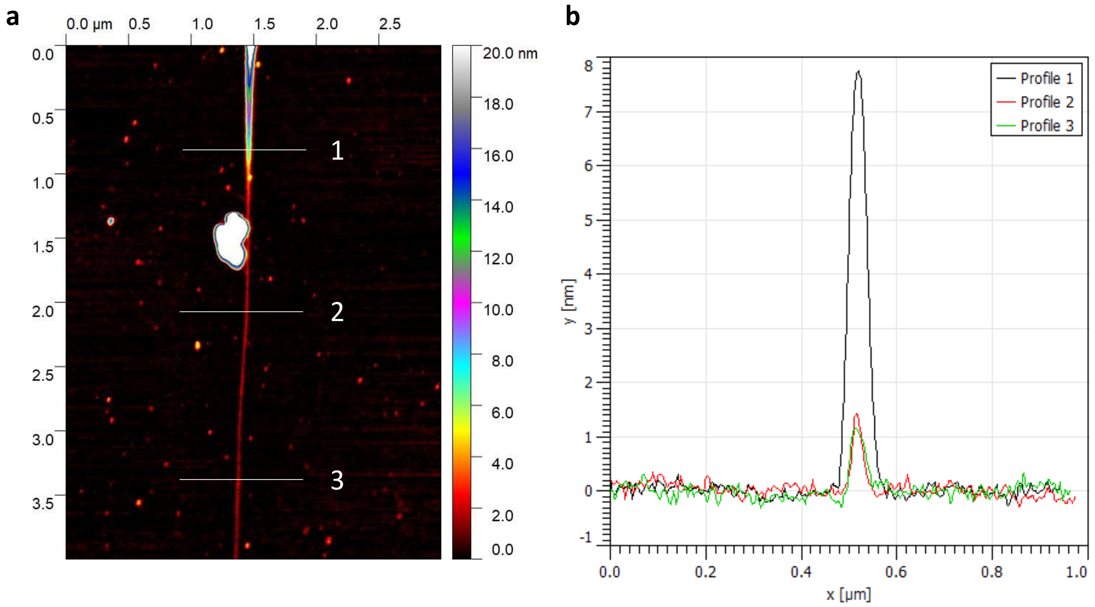

Supplementary Figure 20: Extreme necking of PENF **a**) AFM image of the necked region **b**) Height profiles of lines shown in (**a**).

Cross section of PENFs fabricated by local heating

We have assumed in this work that the fiber cross-section is circular. So, the PENF diameter in the main text was reported based on the width measured using SEM. However, upon measuring the height using atomic force microscopy (AFM), we found that the sample cross section is indeed non-circular at SEM measured widths below 150 nm, where the measured height using

AFM is around 1.5 times smaller than the measured width by SEM, as shown in Supplementary Fig. 21. However, when calculating the k's of the PENFs from the measured thermal conductances, we assumed the fibers to have a cylindrical shape with uniform diameters that correspond to the widths measured by SEM. The same situation is true for σ_{ts} measurements as well. Hence, the k and σ_{ts} reported in the manuscript should be taken as a lower bound for the PENF.

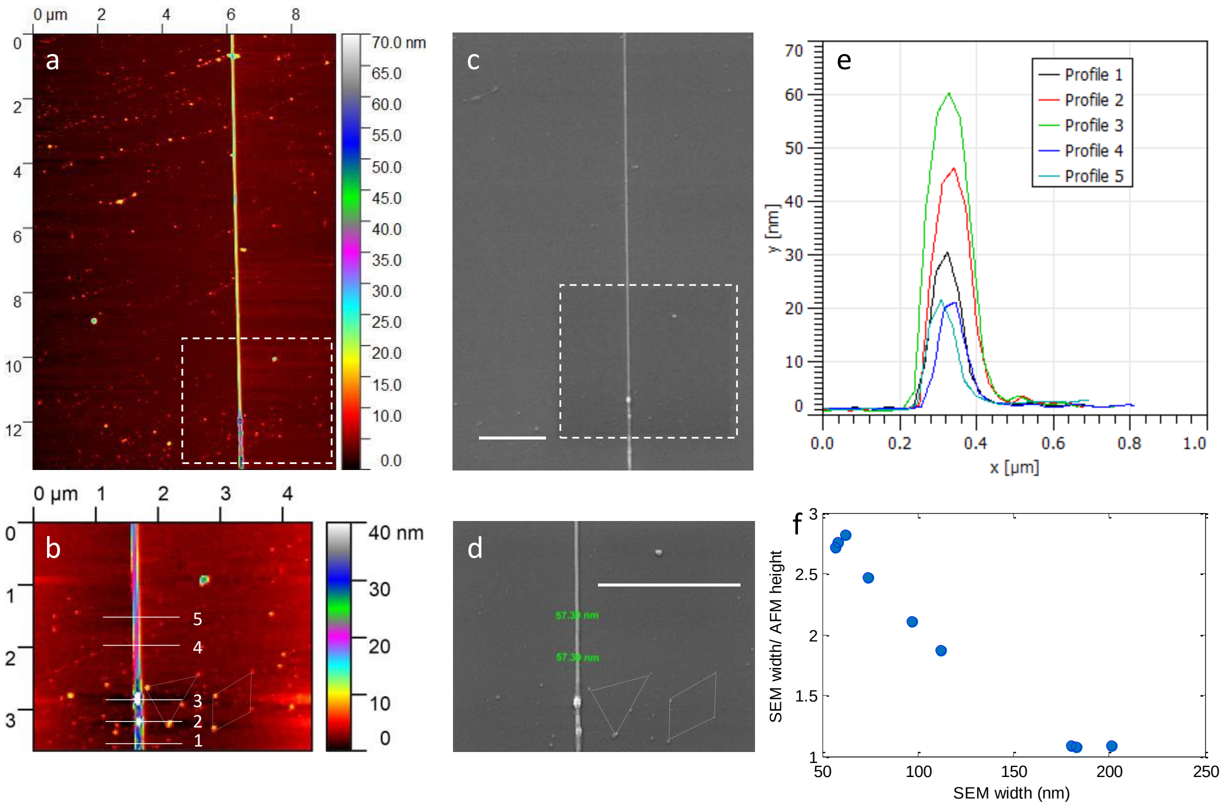

Supplementary Figure 21: Comparison of fiber dimensions measured using AFM and SEM. **a**) Height map measured using AFM. **b**) Zoom in of Inset of (**a**). **c**) Width measured using SEM at same region in (**a**). d) Zoom in inset of (**c**). **e**) Height profile of lines in (**b**). **f**) Ratio of SEM width to AFM height versus SEM width. Scale bars, 2 µm (**c**,**d**).

Shape of a PENF along the length evaluated by SEM

In our k and σ_{ts} measurements, we assume that the PENFs have a cylindrical shape that can be measured by SEM. Supplementary Fig. 22 shows a typical diameter measurement along the fiber length using SEM of a PENF fabricated using local drawing. It can be seen that the diameter is fairly uniform. The sample excludes the dog bone and the tapered region shown in Supplementary Fig. 15. For thermal measurements, since the test length is within 10 μm, we often obtain relatively uniform diameter. Samples with standard deviation of nanofiber diameter larger than 15% are excluded from thermal measurements. However, for mechanical measurements the nominal gage length is 30 μm, so the sample always has some non–uniformity.

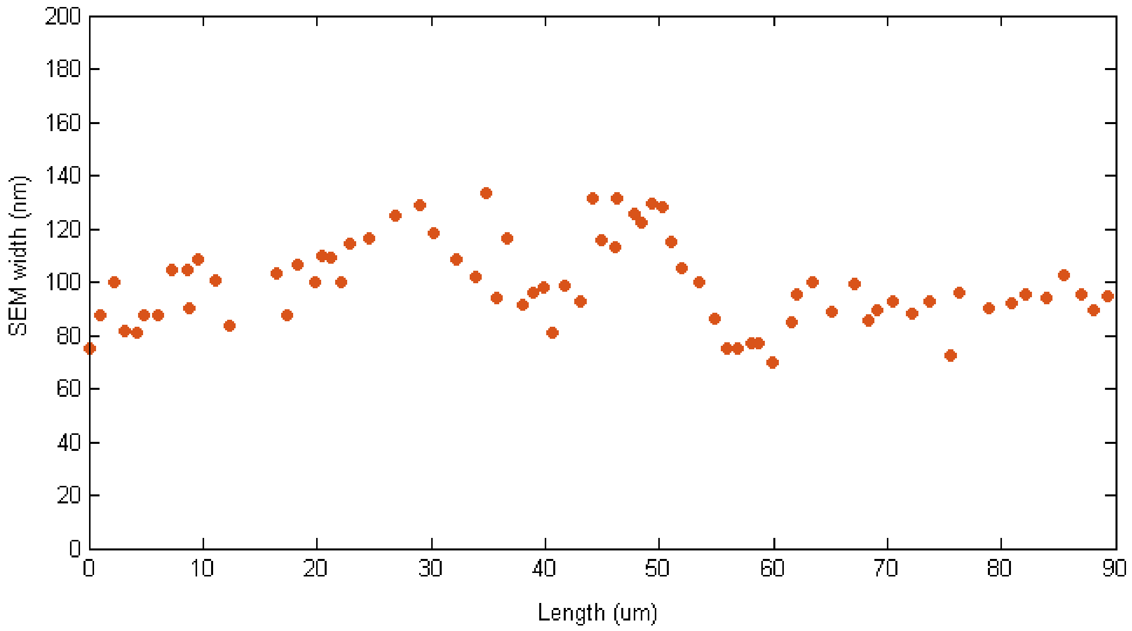

Supplementary Figure 22: Diameter profile of a typical PENF fabricated using local drawing.

Supplementary References

1. Mark, J. E. *Polymer data handbook*. (Oxford University Press, 2009).
2. Park, J. H. & Rutledge, G. C. Ultrafine high performance polyethylene fibers. *J. Mater. Sci.* **53,** (2017).
3. Ma, J. *et al.* Thermal conductivity of electrospun polyethylene nanofibers. *Nanoscale* **7,** 16899–16908 (2015).
4. DSM. Dyneema Fact Sheet. 1–4 (2008). doi:10.2762/41007
5. Zhu, T., Li, J., Ogata, S. & Yip, S. Mechanics of Ultra- Strength Materials Length-Scale Effect.
6. Pop, E., Mann, D., Wang, Q., Goodson, K. & Dai, H. Thermal Conductance of an Individual Single-Wall Carbon Nanotube above Room Temperature. *Nano Lett.* **6,** 96–100 (2006).
7. Pop, E., Varshney, V. & Roy, A. K. Thermal properties of graphene: Fundamentals and applications. *MRS Bull.* **37,** 1273–1281 (2012).
8. Shi, X. M., Bin, Y. Z., Hou, D. S., Men, Y. F. & Matsuo, M. Gelation/crystallization mechanisms of UHMWPE solutions and structures of ultradrawn gel films. *Polym. J.* **46,** 21–35 (2014).
9. Narayanaswamy, A. & Gu, N. Heat Transfer From Freely Suspended Bimaterial Microcantilevers. *J. Heat Transfer* **133,** 42401 (2011).
10. Fujikura, Y., Suzuki, T. & Matsumoto, M. Emissivity of chlorinated polyethylene. *J. Appl. Polym. Sci.* **27,** 1293–1300 (1982).
11. Smith, P. & Lemstra, P. J. Ultrahigh-Strength Polyethylene Filaments by Solution Spinning/Drawing, 2 Influence of Solvent on the Drawability. *Makromol Chem* **180,** 2983–2986 (1979).
12. Sawyer, L. C., Grubb, D. T. & Meyers, G. F. *Polymer Microscopy*. (Springer New York, 2008).
13. Revol, J.-F. & Manley, R. S. J. Lattice imaging in polyethylene single crystals. *J. Mater. Sci. Lett.* **5,** 249–251 (1986).
14. Naraghi, M., Ozkan, T., Chasiotis, I., Hazra, S. S. & de Boer, M. P. MEMS platform for on-chip nanomechanical experiments with strong and highly ductile nanofibers. *J. Micromech. Microeng.*

20, 125022 (2010).

15. Frank, F. C., Keller, A., O ’connor, A. & H. Wills, H. Deformation Processes in Polyethylene Interpreted in terms of Crystal Plasticity. *Phil. Mag.* **3,** (1958).

16. Litvinov, V. M. *et al.* Morphology, Chain Dynamics, and Domain Sizes in Highly Drawn Gel-Spun Ultrahigh Molecular Weight Polyethylene Fibers at the Final Stages of Drawing by SAXS, WAXS, and 1H Solid-State NMR. *Macromolecules* **44,** 9254–9266 (2011).

17. Standard Test Method for Measurement of Enthalpy of Fusion, Percent Crystallinity, and Melting Point of Ultra-High-Molecular Weight Polyethylene by Means of Differential Scanning Calorimetry 1. *ASTM Int. West Conshohocken, PA,* (2016).

18. Strobl, G. R. & Hagedorn, W. Raman spectroscopic method for determining the crystallinity of polyethylene. *J. Polym. Sci. Polym. Phys. Ed.* **16,** 1181–1193 (1978).

19. Shi, L. *et al.* Measuring Thermal and Thermoelectric Properties of One-Dimensional Nanostructures Using a Microfabricated Device. *J. Heat Transfer* **125,** 881 (2003).

20. Li, D. *et al.* Thermal conductivity of individual silicon nanowires. *Appl. Phys. Lett.* **83,** 2934 (2003).

21. Zhu, J. *et al.* Temperature-Gated Thermal Rectifier for Active Heat Flow Control. *Nano Lett.* **14,** 4867–4872 (2014).

22. Yu, C. *et al.* Thermal Contact Resistance and Thermal Conductivity of a Carbon Nanofiber. *J. Heat Transfer* **128,** 234 (2006).

23. Cheng, Z., Liu, L., Xu, S., Lu, M. & Wang, X. Temperature Dependence of Electrical and Thermal Conduction in Single Silver Nanowire. *Sci. Rep.* **5,** 10718 (2015).

24. McGee, G. R., Schankula, M. H. & Yovanovich, M. M. Thermal resistance of cylinder-flat contacts: Theoretical analysis and experimental verification of a line-contact model. *Nucl. Eng. Des.* **86,** 369–381 (1985).

25. Bahadur, V., Xu, J., Liu, Y. & Fisher, T. S. Thermal Resistance of Nanowire-Plane Interfaces. *J. Heat Transfer* **127,** 664 (2005).

26. Choy, C. L., Wong, Y. W., Yang, G. W. & Kanamoto, T. Elastic modulus and thermal conductivity of ultradrawn polyethylene. *J. Polym. Sci. Part B Polym. Phys.* **37,** 3359–3367 (1999).

27. Zihlif, A. M., Duckett, R. A. & Ward, I. M. The Poisson's ratio of ultra-drawn polyethylene and polypropylene fibres using Michelson interferometry. *J. Mater. Sci.* **13,** 1837–1840 (1978).

28. Schwieker, K., Frye, J. & Prorok, B. C. in *MEMS and nanotechnology Volume 6* 41–46 (Springer, New York, NY, 2013).

29. Salvadori, M. C., Brown, I. G., Vaz, A. R., Melo, L. L. & Cattani, M. Measurement of the elastic modulus of nanostructured gold and platinum thin films. *Phys. Rev. B* **67,** 153404 (2003).

30. Israelachvili, J. N. in *Intermolecular and Surface Forces* 253–289 (2011).

31. Batsanov, S. S. Van der Waals Radii of Elements. *Inorg. Mater.* **37,** 871–885 (2001).

32. Neumann, A. W., Omenyi, S. N. & Van Oss, C. J. Negative Hamaker coefficients I. Particle engulfment or rejection at solidification fronts. *Colloid Polym. Sci* **257,** 413–419 (1979).

33. Derjaguin, B. ., Rabinovich, Y. . & Churaev, N. V. Direct measurement of molecular forces. *Nature* **272,** 313–318 (1978).

34. Shen, S., Henry, A., Tong, J., Zheng, R. & Chen, G. Polyethylene nanofibres with very high thermal conductivities. *Nat. Nanotechnol.* **5,** 251–5 (2010).

35. Jensen, B. D., de Boer, M. P., Masters, N. D., Bitsie, F. & LaVan, D. A. Interferometry of actuated microcantilevers to determine material properties and test structure nonidealities in MEMS. *J. Microelectromechanical Syst.* **10,** 336–346 (2001).

36. Sandia. SUMMiT V□ Five Level Surface Micromachining Technology Design Manual. **3.2,** (2012).

37. Owens, D. K. & Wendt, R. C. Estimation of the surface free energy of polymers. *J. Appl. Polym. Sci.* **13,** 1741–1747 (1969).

38. Brewis, D. M. & Briggs, D. Adhesion to polyethylene and polypropylene. *Polymer (Guildf).* **22,** 7–16 (1981).

39. Jana, S., Zhamu, A., Zhong, W. H. & Gan, Y. X. Evaluation of adhesion property of UHMWPE fibers/nano-epoxy by a pullout test. *J. Adhes.* **82,** 1157–1175 (2006).

40. Li, P., Hu, L., McGaughey, A. J. H. & Shen, S. Crystalline polyethylene nanofibers with the theoretical limit of Young's modulus. *Adv. Mater.* **26,** 1065–1070 (2014).

41. Chawla, K. K. in *Composite Materials* 212–251 (Springer New York, 1998).

42. Sanborn, B. & Weerasooriya, T. Tensile Properties of Dyneema SK76 Single Fibers at Multiple Loading Rates Using a Direct Gripping Method. *Conf. Proc. Soc. Exp. Mech. Ser.* **65,** 1–4 (2015).
