# Supplementary material for: Crystalline polymer nanofibers with ultra-high strength and thermal conductivity
Source: Nat Commun. 2018 Apr 25;9:1664. doi: 10.1038/s41467-018-03978-3 (PMC5916895; doi:10.1038/s41467-018-03978-3)
Supplement: Supplementary file 2 — Description of Additional Supplementary Files [file 41467_2018_3978_MOESM2_ESM.pdf]

**Description of Additional Supplementary Files**

**File Name: Supplementary Movie 1**
**Description:** Movie during localized heat drawing of Polyethylne microfiber to nanofiber.
